# Controlled Translocation of Proteins through a Biological Nanopore for Single-Protein Fingerprint Identification

**DOI:** 10.1021/acs.nanolett.4c04510

**Published:** 2024-10-24

**Authors:** Adina Sauciuc, Giovanni Maglia

**Affiliations:** † Groningen Biomolecular Sciences & Biotechnology Institute, University of Groningen, 9747 AG Groningen, The Netherlands

**Keywords:** nanopores, electro-osmosis, free translocation, molecular brakes, protein fingerprinting

## Abstract

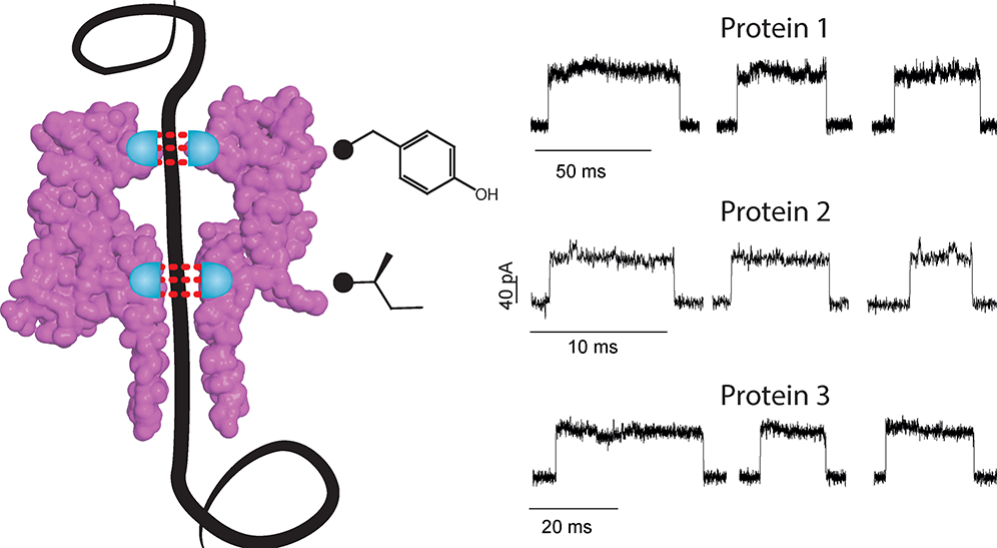

After the successful sequencing of nucleic acids, nanopore technology has now been applied to proteins. Recently, it has been demonstrated that an electro-osmotic flow can be used to induce the transport of unraveled polypeptides across nanopores. Polypeptide translocation, however, is too fast for accurate reading its amino acid compositions. Here, we show that the introduction of hydrophobic residues into the lumen of the nanopore reduces the protein translocation speed. Importantly, the introduction of a tyrosine at the entry of the nanopore and an isoleucine at the entry of the β-barrel of the nanopore reduced the speed of translocation to ∼10 amino acids/millisecond while keeping a relatively large ionic current, a crucial component for protein identification. These nanopores showed unique features within their current signatures, which may pave the way toward protein fingerprinting using nanopores.

Nanopores are under investigation for the single-molecule characterization of proteins. Early work describing protein translocation across nanopores in the presence of guanidinium chloride showed that this process can be very fast, less than a milisecond.^1−3^ Later works, investigating unassisted translocation of proteins across α-hemolysin^4^ and CytK^5^ nanopores achieved translocation times of a few milliseconds.^4,5^ In one of these approaches, where a D10 tag was used to aid the threading of proteins, it was shown that fingerprinting of proteins could be achieved.^4^ Therefore, if translocation can be achieved without the requirement of tagging, unfolded protein translocation may provide a fast and low-cost way to identify proteins.

Mapping the fingerprint pattern to the protein sequence is challenging because it is uncertain which residues contribute to the final electrical signal and because of the short duration of the events. Ideally, the unidirectional transport of the unraveled proteins is defined by a uniform velocity that is slow enough to characterize all amino acids in the protein. The requirements for single-amino-acid recognition depend on several factors, including the sampling and filtering rates of the electronic devices, the speed of polypeptide translocation, and the ionic current difference between the individual features within the nanopore signal. As a good approximation using biological nanopores, individual features should be longer than ∼100 μs and show an ionic current of ∼10 pA.

Recently,^5^ we showed that by introducing charges in the lumen of a nanopore, it is possible to create a strong electro-osmotic flow (EOF) that can mediate the translocation of untagged polypeptides in the presence or absence of denaturants. Although several nanopores can be engineered, we also have shown that to obtain a linearized transport, required for protein fingerprinting or sequencing, the nanopores’ inner lumen should be uniformly narrow (< ∼1.2 nm).^6^ By contrast, nanopores whose lumen has a larger opening favored the formation of structures (blobs^7,8^) within the lumen. Blob formation may be related, among others, to local variations in the speed of the protein. Recent work has shown that the velocity profile of the fluid flow through different nanopores is uneven along the nanopore length, changing based on the nanopore shape and location of the charges.^9^ This factor could further contribute to the uneven velocity of protein translocation.

To decelerate translocation, the EOF could be tuned down, either by changing the buffer composition (ionic strength,^10^ or pH^11^), or by modifying the charges within the nanopore by mutagenesis.^9,12−14^ Alternatively, the EOF could be maintained, but the chemical composition of the nanopore surface could be changed such that additional interactions between the protein and the nanopore lumen would increase the dwell time. Regardless of the strategy, the reduction in the speed of translocation should consider preventing the formation of blobs and allowing a significant large amount of ionic current during the protein blockade.

In this work we use CytK-4D (Figure 1A),^5^ a nanopore whereas the introduction of four aspartate residues allows the linearized translocation of proteins in the presence of urea. We then explore the addition of molecular “brakes” by introducing large, aromatic, or charged amino acids at the contact sites within the CytK-4D nanopore at the *cis* entry (constriction of ∼1.1 nm) and within the β-barrel of the nanopore (constriction of ∼1.2 nm). We found that the engineering of the *cis* entry of the nanopore is attractive, as changes at this site minimally alter the current signature while reducing the translocation speed. Conversely, changes in the barrel that decreased the translocation velocity most often also reduced the ionic current and induced blob formation. Nonetheless, we found that the introduction of the aliphatic residue isoleucine in the β-barrel achieved a reduction of translocation speed without influencing the ionic current or the formation of blobs.

**Figure 1:**
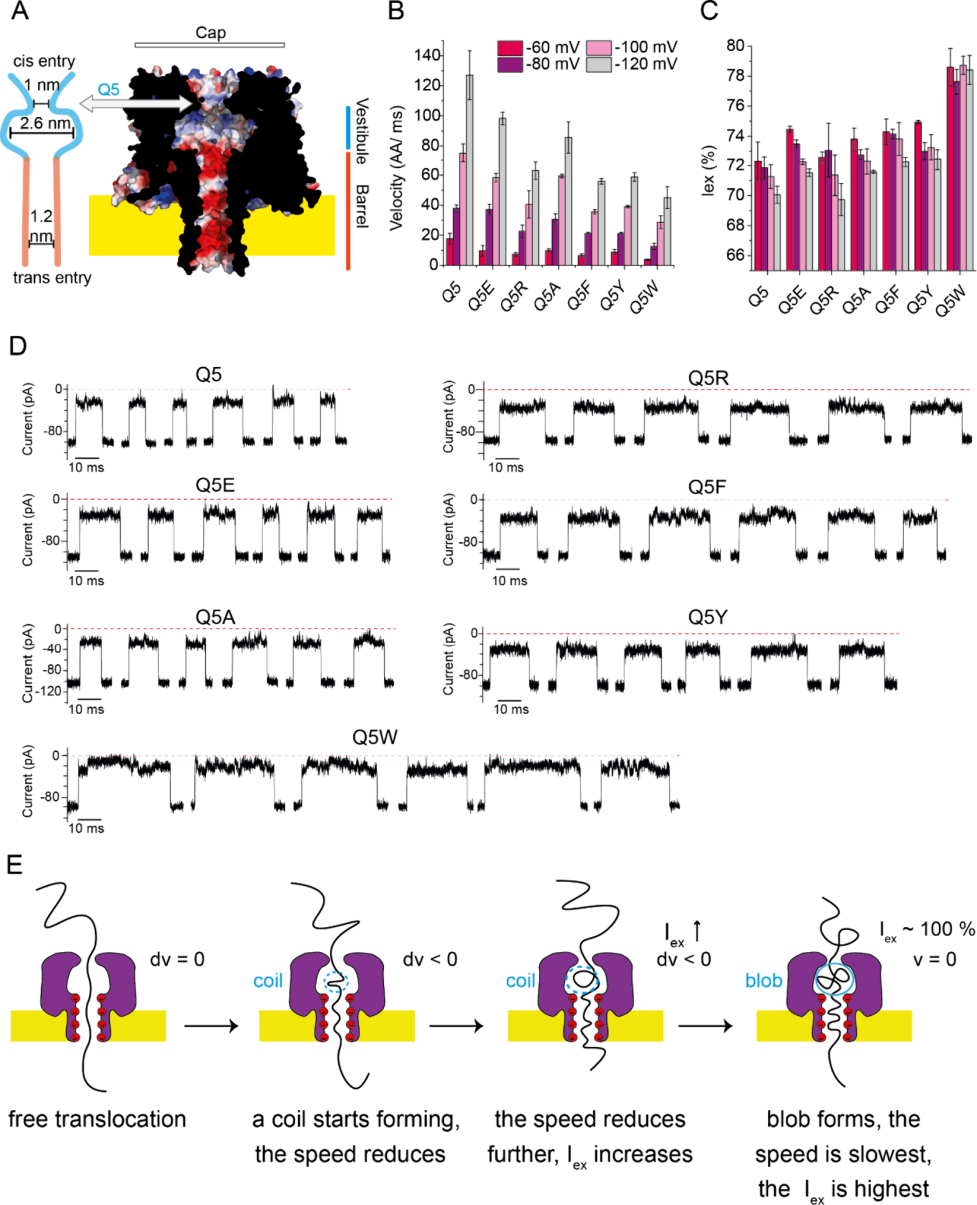
**Control of polypeptide transport by modification of the nanopore *cis* entry**. **(A)** Schematic representation of the lumen (left) and cut through of CytK-4D with the Q5 residue highlighted (right). The pore is shown as a surface representation. **(B–C)** Velocity **(B)** and Iex% (**C**) for the translocation of malE219a across CytK mutants. **(D)** Examples of translocation events at −80 mV form CytK mutants at position 5. **(E)** Depiction of the blob formation mechanism. During free translocation, the velocity of the polypeptide may be constant. When a coil (partially folded structure) starts to form in the vestibule of the nanopore, the velocity of translocation may decrease locally due to adherence to the nanopore walls, and (transient) coils may form. Blob formation may occur, especially within the vestibule, which may further decrease the velocity and result in no residual current/nearly full block. Data was collected from 3 nanopores in 1 M KCl, 15 mM HEPES, 2 M urea, pH 7.5, 50 kHz sampling rate and 10 kHz Bessel filter.

## Hydrophobic Interactions at the Nanopore Entry Decrease the Translocation Velocity

The translocation of an unraveled protein across CytK nanopores was initially sampled using the malE219a substrate in 2 M urea, 1 M KCl, as previously reported.^5^ We used the 2E-4D-CytK nanopore (or simply CytK-4D), which consists of an ∼2.6 nm wide vestibule flanked by a 1.1 nm entry and a β-barrel 1.2 nm wide (Figure 1A). Gln5 is situated at the narrowest nanopore entry point and was replaced with Glu, Arg (charged residues), Ala (small residue), Phe, Tyr, and Trp (aromatic residues; Figure S1–S6). The translocation process was characterized by recording the velocity of polypeptide transport—defined as the ratio between the number of amino acids in the protein and the dwell time of the translocation events (expressed in AAs/ms)—and the excluded current (Iex%)—defined as (I_0_ – I_b_)/I_0_ (%), where I_0_ is the open pore current and I_b_ the blocked pore current.

Compared to the CytK-4D nanopore (velocity = 37.8 ± 2.3 AA/ms, −80 mV), Glu and Ala substitutions resulted in comparable velocities (37.1 ± 3.5, and 30.6 ± 3.7 AA/ms, −80 mV respectively, Figure 1B), suggesting that small changes of steric interactions only have a small influence on the speed of protein translocation. By contrast, the introduction of larger aromatic (Phe, Tyr and Trp) or large and charged (Arg) amino acids induced larger decreases in the translocation velocity (22.8 ± 4.1, 21.2 ± 0.8, 21.1 ± 0.9 and 12.7 ± 2.1 AA/ms, −80 mV respectively, Figure 1C), suggesting that the hydrophobic/hydrophobic, hydrophobic/charged or charged/charged interactions between the translocating polymer and the side chain of the nanopore play a role. In the case of Q5R, the positive charge of arginine likely also weakened the EOF, which might contribute to a reduction of the translocation speed.

Interestingly the substitution Q5Y allowed to sample potentials up to −160 mV (104.5 ± 15.4 AA/ms at −160 mV, Figure S4 and S5) before long-lasting protein blockades prevented protein characterization, while the other nanopores showed a multitude of long-lasting events at potentials higher than −120 mV. Curiously, unfoldases use tyrosine residues to interface with the unfolded polypeptides,^15^ suggesting that the presence of a small chemical group (−OH) can alter the nanopore-protein interactions.

Importantly, at −80 mV, the Iex% remained within 2% of the CytK-4D nanopore (71.9 ± 0.8%, −80 mV), indicating that while the translocation velocity is reduced, a relatively large amount of ion current remains for protein identification. Interestingly, the current blockades showed fluctuations (current signature), which might be specific to the used polypeptide. Each mutation altered the current signature, as shown in scatter plots (Figure S1–S6) and traces (Figure 1D). An exception was CytK-4D-Q5W, which showed a large decrease in velocity (12.7 ± 2.1 AA/ms, −80 mV) associated with a large increase in Iex% (77.6 ± 0.8%, −80 mV). Possibly, the bulky aromatic tryptophan induces a strong interaction with the translocating polypeptide that causes the formation of blobs or the transport of partially folded structures^6−8^ (Figure 1E).

## β-Barrel Modifications Result in Position-Dependent Effects

Having identified CytK-4D-Q5Y as the nanopore that decreases the velocity 2-fold, while minimally impacting Iex%, we proceeded with exploring β-barrel modifications. Modifications at this site may alter the current signature (protein ID) based on the intrinsic properties, such as location and (local) content of aromatic residues within the protein. A Phe residue within the β-barrel of CytK and aerolysin nanopores was shown to facilitate peptide detection and discrimination.^16^ However, recent work^6^ showed that placing a Phe residue at the bottom of the β-barrel (S126F) had adverse effects on polypeptide translocation, causing blob formation and substantially increasing the blocked current during translocation (Iex% from 71.9 ± 0.8% to 88.7 ± 0.3% at −80 mV). Nevertheless, we examined whether the impact of Phe changed with its depth in the β-barrel by using the CytK-4D nanopore. We mutated several residues at various distances (*d*) from the entry of the barrel (Cα X to Cα E112)(Figures S7–S11).

**Figure 2:**
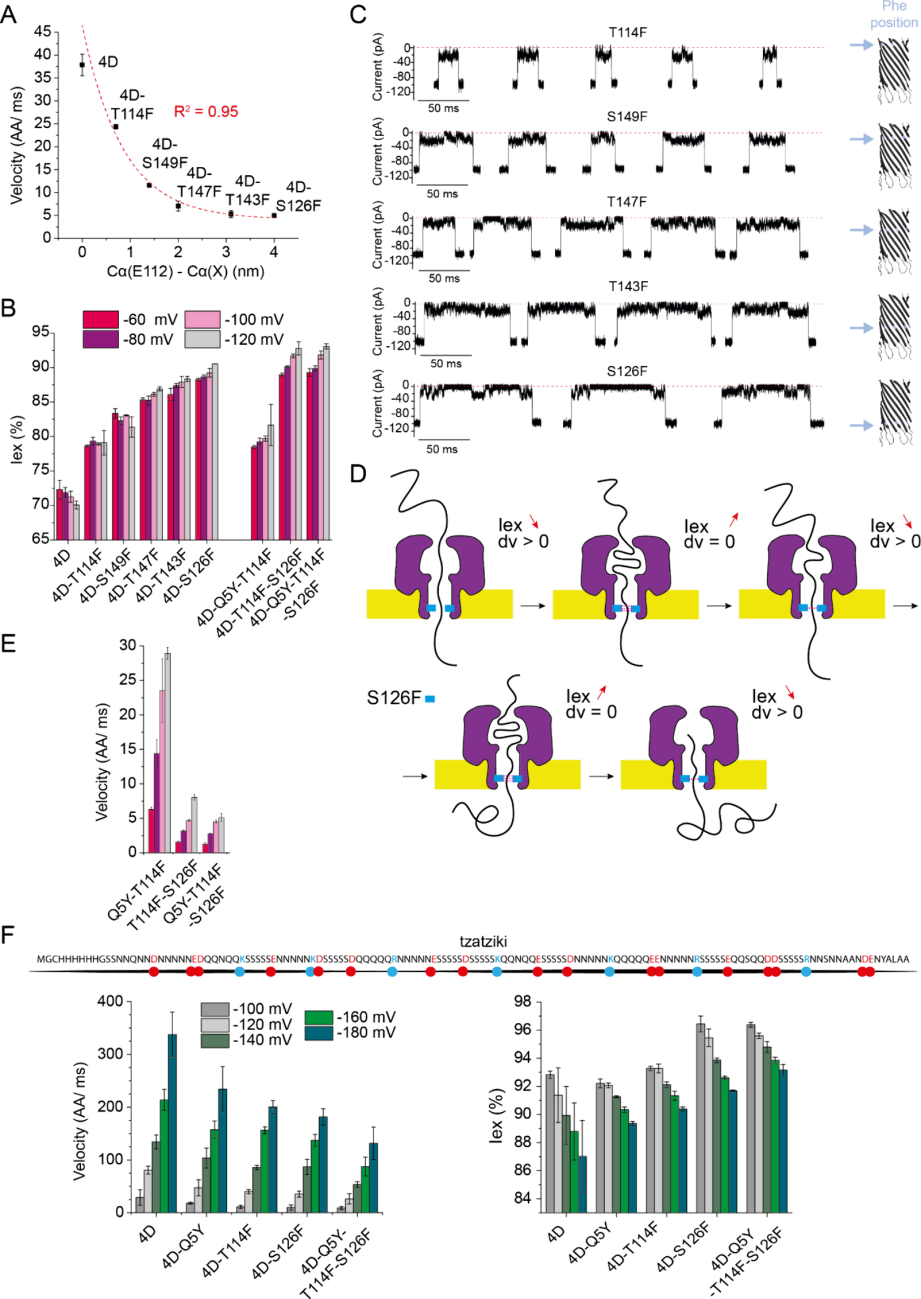
**Introduction of Phe in CytK-4D β-barrel. (A)** Exponential fit of the velocity from the single Phe mutants introduced in the β-barrel of CytK-4D. **(B)** Iex% from all aromatic mutants. **(C)** Examples of translocation events from mutants bearing one Phe residue. **(D)** Potential mechanism of S126F halting polypeptide translocation due to local interactions causing sudden changes in velocity. **(E)** Velocity from mutants with multiple aromatic residues in the lumen. **(F)** Tzatziki, a model substrate devoid of aromatic residues, and the velocity and Iex% from several mutants with aromatic residues. Data was collected from 3 nanopores in 1 M KCl, 15 mM HEPES, 2 M urea, pH 7.5, 50 kHz sampling rate and 10 kHz Bessel filter for malE219a. The same conditions, excluding urea, were used for tzatziki.

The velocity of polypeptide translocation decreased exponentially with the distance from the barrel entrance: T114F (24.4 ± 0.5 AA/ms, *d* = 0.7 nm) > S149F (11.6 ± 0.3 AA/ms, 1.4 nm) > T147F (7.0 ± 1.1 AA/ms, 2.0 nm) > T143F (5.3 ± 0.8 AA/ms, 3.1 nm) > S126F (4.9 ± 0.4 AA/ms, 4.0 nm) across all tested potentials (Figures 2A, Figure S12). Phe substitutions also altered the Iex% substantially. On one hand, Iex% increased proportionally with the depth of the residue: from 79.3 ± 0.5% for T114F (0.7 nm) to 88.7 ± 0.3% for S126F (4.0 nm), as shown in Figure 2B. In the case of T147F, T143F and S126F, the Iex% increased with the applied potential, as opposed to CytK-4D, suggesting that the phenylalanine residues also increased blob formation.

A close inspection of the current signatures from each mutant (Figure 2C) gives insight into the potential mechanism of deceleration. The signatures from the deeper rings, T143F (*d* = 3.1 nm) and S126F (*d* = 4.0 nm), comprise full blockades alternating with open blockades. On the other hand, the shallower rings, T114F (*d* = 0.7 nm) and S149F (*d* = 1.4 nm), reveal nonuniform patterns, without reaching a full-block level. Finally, the middle ring, T147F (*d* = 2.0 nm), shares similarities with both pairs: a full-block level is occasionally reached within a nonuniform signature pattern. A possible explanation is depicted in Figure 2D. The interaction between the translocating polypeptides and the introduced aromatic residue likely introduces local disruptions in the translocation velocity. These sudden “brakes” on the polypeptide movement in the β-barrel region may result in the translocating polymer coiling up within the vestibule and the formation of blob/coil. As the formation of blobs reduces the ion flow, the electro-osmotic force is also reduced, which then results in an increase in dwell time without gaining significant improvements in resolution/feature detection (Figure 2D). Since the strongest electro-osmotic flow is at the constriction of the β-barrel region, the further the aromatic brakes are from the entry of the β-barrel region, the higher the probability of blob formation is.

## Dual Mechanism in Decelerating Translocation

Although phenylalanine residues slowed down the translocation of unfolded polypeptides, they also increased the excluded current, both being disadvantageous for fingerprint analysis. We reasoned that the deceleration in polypeptide translocation is most possibly caused by polypeptide-nanopore hydrophobic interactions. To test this hypothesis, we constructed nanopores with multiple aromatic interactions in both the entry and barrel (Q5Y-T114F, T114F–S126F and Q5Y-T114F–S126F substitutions in the CytK-4D nanopore) and tested with substrates with aromatic residues (urea-destabilized malE219a) and a model substrate almost devoid of aromatic residues.

We found that the translocation of malE219a is progressively decelerated as more aromatic residues are introduced into the lumen: 37.8 ± 2.3 AA/ms for CytK-4D, 14.3 ± 2.0 AA/ms for CytK-Q5Y-T114F, 3.1 ± 0.1 AA/ms for CytK-T114F–S126F, and 2.7 ± 0.1 AA/ms for CytK-Q5Y-T114F–S126F at −80 mV (Figure 2E, Figures S13–S15). On the other hand, the excluded current also increased: 71.9 ± 0.8% for CytK-4D, 79.2 ± 0.5% for CytK-4D-Q5Y-T114F, 90.1 ± 0.2% for CytK-4D-T114F–S126F, and 89.9 ± 0.4% for CytK-4D-Q5Y-T114F–S126F at −80 mV (Figure 2B). The increase in Iex% was also associated with blob formation during translocation (Figure S15).

By contrast, tzatziki, a model polypeptide almost completely devoid of aromatic residues (contains one Tyr), traversed the CytK-4D-Q5Y, CytK-4D-T114F, CytK-4D-S126F and CytK-4D-Q5Y-T114F–S126F mutants, resulting in substantially different trends for both the velocity and Iex% (Figure 2F, S16–S19). First, the velocity decreased less abruptly among the single mutants: 134.2 ± 13.0 AA/ms for CytK-4D, 103.7 ± 19.1 AAs/ms for CytK-4D-Q5Y, 85.7 ± 3.9 AA/ms for CytK-4D-T114F, 87.0 ± 14.5 AA/ms for CytK-4D-S126F and 53.5 ± 5.5 AAs/ms for CytK-4D-Q5Y-T114F–S126F mutant at −140 mV. Second, the Iex% dependence on the applied potential was retained across all mutants (Figure 2F).

We conclude that aromatic amino acids decelerate the polypeptide translocation through a combination of friction, defined here as the steric interactions between the nanopore and the translocating polypeptide, and aromatic adhesion, defined here as the interaction between the aromatic residues in the nanopore and the translocating peptides. In the case of the translocation of the model polypeptide tzatziki, which is almost devoid of aromatic amino acids, translocation may only be affected by the friction. This may explain the relatively constant translocation velocity and reduced formation of blobs compared to unravelled proteins. Proteins, on the other hand, have aromatic residues scattered within their primary sequence. Thus, it is likely that hydrophobic interactions between the engineered aromatic residues within the nanopore and the polypeptide promote sudden local decelerations in the polypeptide translocation speed, which might propagate through the entire polypeptide chain. This would eventually result in transient halting in movement and coil or blob formation (Figure 2D). Therefore, although it is possible to reduce the speed of translocation substantially by introducing aromatic amino acids at the *cis* constriction and in the β-barrel region, this comes at a cost in terms of Iex% and blob formation.

## A Smaller Hydrophobic Amino Acid within the β-Barrel

**Figure 3:**
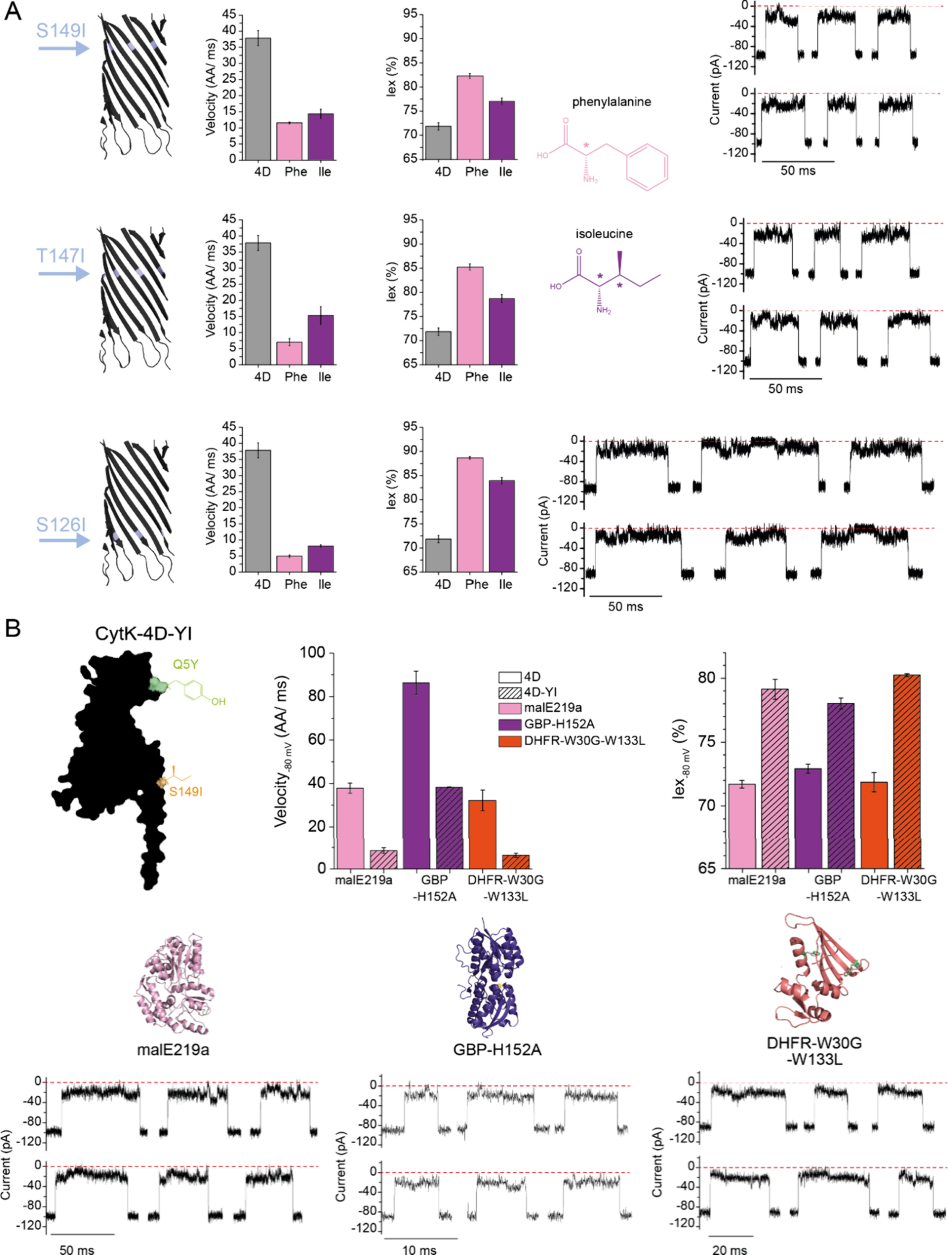
**Introduction of Ile at the β-barrel level**. **(A)** Ile introduction at three positions in the β-barrel, S149 (top), T147 (middle), and S126 (bottom) and their effect on the velocity, Iex%, and the current signature in the translocation of malE219a. The structures of Phe and Ile are depicted, with the chiral centers highlighted with (*). **(B)** Translocation of three proteins, malE219a, GBP-H152A and DHFR-W30G-W133L across the CytK-4D-Q5Y-S149I (CytK-4D-YI). Data was acquired from 3 nanopores in 1 M KCl, 15 mM HEPES, 2 M urea (malE219a) or 2.6 M urea (GBP and DHFR), pH 7.5, −80 mV, 50 kHz sampling rate, and 10 kHz Bessel filter.

To separate chemical and steric effects, we introduced a small hydrophobic residue, isoleucine, whose aliphatic side chain is slightly larger than the one of serine and threonine, along with a chiral center, potentially relevant for fingerprinting or PTM detection. We tested three isoleucine substitutions: S126I, T147I and S149I within the CytK-4D nanopore (Figure 3A, S20–S22). Residues located in the middle and top of the β-barrel reduced polypeptide translocation velocity by ∼2-fold (e.g., 15.3 ± 2.8 and 14.4 ± 1.5 AAs/ms, for T147I and S149I, respectively, at −80 mV). For these residues, Iex% slightly increased (78.7 ± 0.8% and 77.0 ± 0.7% at −80 mV, respectively) compared to CytK-4D (71.9 ± 0.8%). S126I, located deepest within the barrel, resulted in a more substantial velocity reduction (8.1 ± 0.4 AAs/ms at −80 mV); however, Iex% also increased (83.9 ± 0.7%). Furthermore, for S126I several full blockages within the translocation events were observed (Figure 3A).

To obtain a nanopore that would reduce the translocation speed while keeping a large ionic current and minimizing the formation of blobs, we constructed CytK-4D-Q5Y-S149I mutant (CytK-4D-YI in short), which combines a mutation at the entry of the nanopore and one in the β-barrel. In 2.6 M urea, this nanopore obtained a translocation velocity of 8.5 AAs/ms (−80 mV), while maintaining a relatively low Iex% (79.1 ± 0.8%, Figure 3B, S23). CytK-4D-YI was tested with two other native-like substrates: GBP-H152A and DHFR-W30G-W133L (Figure 3B). Notice that compared to a previous report^5^ the GBP variant used here did not include a periplasmic tag (ANKKVITLSAVMASMLFGAAAHA, Figure S24). Compared to malE219a, both proteins showed a similar average Iex% (Figure 3B). While DHFR was transported at a similar velocity (6.4 ± 1.0 AAs/ms at −80 mV) GBP translocation was about 6-fold faster (38.2 ± 0.2 AAs/ms at −80 mV, Figure 3B, Figure S25, S26). These differences may arise from the charge densities of the proteins (−1.76_100_, −6.06_100_ and −2.37_100_, for GBP, DHFR and malE219a, expressed as number of net charge per 100 amino acids, respectively), or the aromatic content (expressed as % of Phe, Tyr and Trp, 5.87%, 7.65% and 9.71%, respectively), or a combination thereof.

In this work, we explored whether unassisted polypeptide translocation can be decelerated by engineering the CytK-4D nanopore at multiple sites. Large residues introduced at the *cis* constriction reduced the translocation speed compared to CytK-4D while maintaining large ionic currents during the blockades. We found that the introduction of a tyrosine residue was particularly favorable, because it also allowed sampling at significantly higher potentials (up to −160 mV compared to −120 mV in other pores). Highly conserved Tyr residues are found in the inner loops of unfoldases,^15^ where they aid substrate progression by intercalating with the substrate and pointing their hydroxyl toward the peptide bond. CytK-4D-Q5Y may have a similar role in polypeptide translocation across the CytK nanopore, potentially further assisting linearization at the nanopore entry, next to decreasing the velocity.

The introduction of aromatic residues into the β-barrel induced a strong decrease in the translocation velocity. Interestingly, the latter decreased exponentially with the distance of the aromatic amino from the *cis* side. However, these substitutions also increased the likelihood of blob formation and strongly increased the percentage of blocked current. By contrast, the introduction of an aliphatic residue, such as isoleucine, resulted in a better compromise between reducing the translocation velocity, preventing blob formation, and maintaining the Iex% for protein recognition.

In particular, the CytK-4D-Q5Y-S149I mutant showed a 60–80% reduced velocity compared to CytK-4D while maintaining a similar Iex%. Although the degree of deceleration achieved with the same nanopore varied with the different tested protein substrates depending on their charge and aromatic content, a translocation speed of less than 10 amino acid per ms was observed for urea destabilized malE219a and DHFR-W30G-W133L, a speed that is compatible with single-amino-acid resolution.

## Data Availability

Raw data was deposited at 10.5281/zenodo.13383283.

## Supporting Information Available

Additional information on the translocation of the protein of interest through the various mutant nanopores (PDF)

## References

[ref1] Pastoriza-Gallego M.; et al. Dynamics of Unfolded Protein Transport through an Aerolysin Pore. J. Am. Chem. Soc. 2011, 133, 2923–2931. 10.1021/ja1073245. 21319816

[ref2] Pastoriza-Gallego M.; et al. Evidence of Unfolded Protein Translocation through a Protein Nanopore. ACS Nano 2014, 8, 11350–11360. 10.1021/nn5042398. 25380310

[ref3] Rodriguez-Larrea D.; Bayley H. Protein co-translocational unfolding depends on the direction of pulling. Nat. Commun. 2014, 5, 4841 10.1038/ncomms5841. 25197784 PMC4164780

[ref4] Yu L.; et al. Unidirectional single-file transport of full-length proteins through a nanopore. Nat. Biotechnol. 2023, 41, 1130 10.1038/s41587-022-01598-3. 36624148 PMC10329728

[ref5] Sauciuc A.; Morozzo della Rocca B.; Tadema M. J.; Chinappi M.; Maglia G. Translocation of linearized full-length proteins through an engineered nanopore under opposing electrophoretic force. Nat. Biotechnol. 2024, 42, 1275 10.1038/s41587-023-01954-x. 37723268

[ref6] Sauciuc A. Blobs form during the single-file transport of proteins across nanopores. Proc. Natl. Acad. Sci. U. S. A. 2024, 121, e2405018121 10.1073/pnas.2405018121. 39264741 PMC11420176

[ref7] Daoud M.; De Gennes P. G. Statistics of macromolecular solutions trapped in small pores. J. Phys. (Paris) 1977, 38, 85–93. 10.1051/jphys:0197700380108500.

[ref8] Cressiot B.; et al. Dynamics and Energy Contributions for Transport of Unfolded Pertactin through a Protein Nanopore. ACS Nano 2015, 9, 9050–9061. 10.1021/acsnano.5b03053. 26302243 PMC4835817

[ref9] Li M.; Muthukumar M. Electro-osmotic flow in nanoconfinement: Solid-state and protein nanopores. J. Chem. Phys. 2024, 160, 084905 10.1063/5.0185574. 38411234

[ref10] Wong C. T. A.; Muthukumar M. Polymer capture by electro-osmotic flow of oppositely charged nanopores. J. Chem. Phys. 2007, 126, 164903 10.1063/1.2723088. 17477630

[ref11] Asandei A.; et al. Electroosmotic Trap Against the Electrophoretic Force Near a Protein Nanopore Reveals Peptide Dynamics During Capture and Translocation. ACS Appl. Mater. Interfaces 2016, 8, 13166–13179. 10.1021/acsami.6b03697. 27159806

[ref12] Huang G.; Willems K.; Soskine M.; Wloka C.; Maglia G. Electro-osmotic capture and ionic discrimination of peptide and protein biomarkers with FraC nanopores. Nat. Commun. 2017, 8, 935 10.1038/s41467-017-01006-4. 29038539 PMC5715100

[ref13] Huang G.; et al. Electro-Osmotic Vortices Promote the Capture of Folded Proteins by PlyAB Nanopores. Nano Lett. 2020, 20, 3819–3827. 10.1021/acs.nanolett.0c00877. 32271587 PMC7227020

[ref14] Gubbiotti A. Electroosmosis in nanopores: computational methods and technological applications. Adv. Phys. X 2022, 7, 2036638 10.1080/23746149.2022.2036638.

[ref15] Martin A.; Baker T. A.; Sauer R. T. Pore loops of the AAA+ ClpX machine grip substrates to drive translocation and unfolding. Nat. Struct Mol. Biol. 2008, 15, 1147–1151. 10.1038/nsmb.1503. 18931677 PMC2610342

[ref16] Versloot R. C. A.; Straathof S. A. P.; Stouwie G.; Tadema M. J.; Maglia G. β-Barrel Nanopores with an Acidic-Aromatic Sensing Region Identify Proteinogenic Peptides at Low pH. ACS Nano 2022, 16, 7258–7268. 10.1021/acsnano.1c11455. 35302739 PMC9134492

