# Supplementary material for: Controlled Translocation of Proteins through a Biological Nanopore for Single-Protein Fingerprint Identification
Source: Nano Lett. 2024 Oct 24;24(44):14118–24. doi: 10.1021/acs.nanolett.4c04510 (PMC11544688; doi:10.1021/acs.nanolett.4c04510)
Supplement: Supplementary file 1 — nl4c04510_si_001.pdf [file nl4c04510_si_001.pdf]

Supporting information

**Controlled translocation of proteins through a biological nanopore for single-protein fingerprint identification**

Adina Sauciuc and Giovanni Maglia

[1]Groningen Biomolecular Sciences & Biotechnology Institute, University of Groningen, 9747 AG Groningen, The Netherlands

**Table of contents**

Materials and Methods

Tables dwell time, velocity and excluded current CytK mutants + malE219a

Tables dwell time, velocity and excluded current CytK mutants + tzatziki

Tables dwell time, velocity and excluded current CytK Q5Y-S149I + GBP or DHFR proteins

Figure S1 Translocation of malE219a through the CytK 4D-Q5E

Figure S2 Translocation of malE219a through the CytK 4D-Q5R.

Figure S3 Translocation of malE219a through the CytK 4D-Q5A

Figure S4 Translocation of malE219a through the CytK 4D-Q5F

Figure S5 Translocation of malE219a through the CytK 4D-Q5Y

Figure S6 Translocation of malE219a through the CytK 4D-Q5W

Figure S7 Translocation of malE219a through the CytK 4D-T114F nanopore

Figure S8 Translocation of malE219a through the CytK 4D-S126F nanopore

Figure S9 Translocation of malE219a through the CytK 4D-T143F nanopore

Figure S10 Translocation of malE219a through the CytK 4D-T147F nanopore

Figure S11 Translocation of malE219a through the CytK 4D-S149F nanopore

Figure S12 Exponential fits of the velocities (AAs/ ms) through the CytK-4D-X->F mutants

Figure S13 Translocation of malE219a through the CytK 4D-Q5Y-T114F nanopore

Figure S14 Translocation of malE219a through the CytK 4D-T114F-S126F nanopore

Figure S15 Translocation of malE219a through the CytK 4D-Q5Y-T114F-S126F nanopore

Figure S16 Translocation of tzatziki through the CytK 4D-Q5Y nanopore

Figure S17 Translocation of tzatziki through the CytK 4D-T114F nanopore

Figure S18 Translocation of tzatziki through the CytK 4D-S126F nanopore.

Figure S19 Translocation of tzatziki through the CytK 4D-Q5Y-T114F-S126F nanopore

Figure S20 Translocation of malE219a through the CytK 4D-S126I nanopore.

Figure S21 Translocation of malE219a through the CytK 4D-T147I nanopore.

Figure S22 Translocation of malE219a through the CytK 4D-S149I nanopore

Figure S23 Translocation of malE219a through the CytK 4D-Q5Y-S149I nanopore.

Figure S24 Translocation of GBP-H152A Δperi through the CytK 4D nanopore

Figure S25 Translocation of GBP-H152A Δperi through the CytK 4D-Q5Y-S149I nanopore

Figure S26 Translocation of DHFR-W30G-W133L through the CytK 4D-Q5Y-S149I nanopore

## Chemicals

The chemicals and suppliers used are listed as follows: Ampicillin sodium salt was purchased from Fisher Bio Reagents; chloramphenicol (≥98.0) from Sigma Life Science); urea (≥99.5%), guanidinium chloride (≥99.5%, biochemistry), Isopropyl β-D-thiogalactopyranoside (≥99.0%, dioxin-free, animal-free), LB medium, 2xYT medium, NaCl (≥99.5%), HEPES (PUFFERAN® CELLPURE® (≥99.5%), imidazole (≥99%), KCl (≥99.5%), Tris(2-carboxyethyl)phosphine hydrochloride (≥98.0%), Dodecyl-β-D-maltoside (≥99%) from Roth; and n-hexadecane (99% from Acros Organics; protease inhibitors (Pierce™ Protease inhibitor Mini tablets, EDTA-free); GeneJET gel extraction kit, GeneJET PCR purification kit, GeneJET Plasmid Miniprep kit were purchased from (Thermo Scientific); Ni-NTA agarose from Qiagen; Strep Tactin® Sepharose® and D-desthiobiotin from IBA Lifesciences; DPhPC from Avanti polar lipids, n-pentane from Sigma-Aldrich; Quick Start Bradford 1x Dye Reagent (Bio-Rad); DNA primers and gBlock™ from IDT. BL21(DE3) strain harbouring the pET-PfuX7 plasmid was kindly provided by prof. dr. Oscar Kuipers.

## Methods

Protein sequences

CytK 4D

MAQTTSQVVTDIGQNAKTHTSYNTFNNEQADNMTMSLKVTFIDDPSADKQIAVINTTGSFMKANPTLSDAPVDG
YPIPGASVTLRYPSQYDIAMNLQDNTSRFFHVAPTNAVEETTVTSSVSYQLGGSIDASVTPSGPSGESGATGDVTWS
DDVSYDQTSYKTNLIDQTNKHVKWNVFFNGYNNQNWGIYTRDSYHALYGNQLFMYSRTYPHETDARGNLVPMN
DLPTLTNSGFSPGMIAVVISEKDTEQSSIQVAYTKHADDYTLRPGFTFGTGNWVGNNIKDVDQKTFNKSFVLDWKN
KKLVEKKGSAHHHHHH

malE219a

MGSSHHHHHHSSGLVPRGSHNKIEEGKLVIWINGDKGYNGLAEVGKKFEKDTGIKVTVEHPDKLEEKFPQVAATG
DGPDIIFWAHDRFGGYAQSGLLAEITPDKAFQDKLYPFTWDAVRYNGKLIAYPIAVEALSLIYNKDLLPNPPKTWEEI
PALDKELKAKGKSALMFNLQEPYFTWPLIAADGGYAFKYENGKYDIKDVGVDNAGAKAGLTFLVDLIKNKHMNAD
TDYSIAEAAFNKDPTAMTINGPWAWSNIDTSKVNYGVTVLPTFKGQPSKPFVGVLSAGINAASPNKELAKEFLENYL
LTDEGLEAVNKDKPLGAVALKSYEEELAKDPRIAATMENAQKGEIMPNIPQMSAFWYAVRTAVINAASGRQTVDE
ALKDAQTNSSSNNNNNNNNNNLGIEGLYFQSH

tzatziki

MGCHHHHHHGSSNNQNNDNNNNNEDQQNQQKSSSSSENNNNNKDSSSSSDQQQQQRNNNNNESSSSSDSS
SSSKQQNQQESSSSSDNNNNNKQQQQQEENNNNNRSSSSSEQQSQQDDSSSSSRNNSNNAANDENYALAA

GBP-H152A

MADTRIGVTIYKYDDNFMSVVRKAIEQDAKAAPDVQLLMNDSQNDQSKQNDQIDVLLAKGVKALAINLVDPAAA
GTVIEKARGQNVPVVFFNKEPSRKALDSYDKAYYVGTDSKESGIIQGDLIAKHWAANQGWDLNKDGQIQFVLLKGE
PGAPDAEARTTYVIKELNDKGIKTEQLQLDTAMWDTAQAKDKMDAWLSGPNANKIEVVIANNDAMAMGAVEAL
KAHNKSSIPVFGVDALPEALALVKSGALAGTVLNDANNQAKATFDLAKNLADGKGAADGTNWKIDNKVVRVPYV
GVDKDNLAEFSKKGSSHHHHHH

DHFR-cc W30G W133L

MAHHHHHHGSAMISLIAALAVDRVIGMENAMPWNLPADLAGFKRNTLDKPVIMGRHTWESIGRPLPGRKNIILSS
QPGTDDRVTWVKSVDEAIAAAGDVPEIMVIGGGRVYEQFLPKAQKLYLTHIDAEVEGDTHFPDYEPDDLESVFSEF
HDADAQNSHSYSFEILERR

**Cloning of the CytK nanopore mutants**

We constructed plasmids containing mutant CytK nanopores through USER cloning. Fragments for the cloning were generated using PfuX7 DNA polymerase as previously described[1]. Homology regions of 8-13 base pairs were identified around the ATG codon, the mutation site, and downstream of the stop codon to facilitate joining of the empty vector with the gene fragments. The full insert was divided into upstream and downstream fragments at the mutation site and amplified with uracil-containing primers. The empty pT7-SC1 backbone (AmpR) was linearized separately using uracil-containing primers. PCR conditions followed those described by Norholm[2], with the extension time reduced from 1 minute per kilobase to 30 seconds per kilobase. Purification of PCR products was carried out either by gel extraction in the presence of by-products or by cleaning up from the PCR mix.

In the USER reaction, the upstream (F1) and downstream (F2) fragments were combined with the empty vector (V) in a molar ratio of 3:3:1. The reaction proceeded as described previously[3]: 25 minutes at 37°C, followed by a 10-minute incubation at 60°C. The mixture was then cooled to room temperature (22 or 20°C) for 15 minutes and stored on ice or at 4°C until the transformation step.

The annealed fragments resulting in circularized plasmids were transformed into chemically competent E. coli cells using the heat shock method (42°C for 70 seconds followed by incubation on ice for 10 minutes). The transformed cells were selected on LB-agar plates supplemented with 100 µg/mL ampicillin and 1% glucose. Plasmids from individual colonies were isolated, and the presence of mutations was confirmed by Sanger sequencing (Macrogen/Eurofins).

**Expression and purification of the CytK nanopores**

The plasmids containing the nanopores were introduced into BL21(DE3) electrocompetent cells via electroporation using a Bio-Rad Micro Pulser with the bacterial setting. Afterward, the cells were plated on media supplemented with 100 µg/mL ampicillin and 1% glucose and were incubated overnight at 37°C. Subsequently, a number of transformants were suspended in 100 mL LB medium supplemented with 100 µg/mL ampicillin, aiming for an initial optical density at 600 nm ($OD_{600}$) between 0.05 and 0.1. These cultures were then grown at 37°C and 180 RPM until reaching an $OD_{600}$ of 0.6-0.8. Upon reaching this point, protein expression was induced by adding 0.5 mM IPTG after chilling the cultures on ice for 5-10 minutes. After an incubation of 19-21 hours at 25°C and 180 RPM, the cells were harvested by centrifugation at 7500 rpm for 5 minutes and the resulting cell pellets were stored at -80°C for at least 30 minutes prior to purification.

Pellets from 100 mL cultures were resuspended in 20-25 mL of ice-cold lysis buffer. Subsequent steps were carried out at 4-6°C unless specified otherwise. The cell suspension was sonicated using a Branson Sonifier 450 at 25% duty cycle and 2.5 output control for 2-3 minutes. After sonication, cellular debris was removed by centrifugation at 8000 RPM for 20 minutes. The resulting supernatant was then incubated with 200 µL of $Ni^{2+}$-NTA slurry (50% suspension) that had been pre-equilibrated and prewashed with 1 mL of lysis buffer for 20-40 minutes with shaking. The beads were briefly pelleted at 1500 RPM for 1 minute and then transferred to a column (2 mL bed volume bio-spin chromatography, BioRad) while allowing the flow-through to pass at room temperature. The column was washed in four steps with a total of 10 mL of wash buffer. Finally, the protein was eluted in three fractions of 150 µL each using elution buffer. The presence of SDS-stable CytK mutant oligomers was confirmed by SDS-PAGE, without the heating step in the sample preparation. The elution fractions containing the CytK nanopores were stored at 4 °C for several months.

The substrate proteins were prepared as previously described[4]. The N-terminal periplasmic sequence was removed from the GBP H152A insert by means of USER cloning.

**Electrophysiology experiments**

Planar lipid bilayer recordings were conducted using a chamber consisting of two compartments separated by a 25 µm thick Teflon membrane containing an aperture of approximately 100 µm, as previously described[5]. A droplet, equivalent to half the volume of a 10 µL glass capillary, containing n-hexadecane dissolved in n-pentane (6.25%) was applied onto the Teflon membrane. Upon evaporation of the n-pentane, 500 µL of buffer solution was added, followed by two-three droplets of DPhPC lipids in n-pentane (5 mg/mL) in each compartment. Ag/AgCl electrodes were connected to the two compartments via 2.5% agarose bridges containing a 3M KCl solution, grounding the cis compartment. Recordings were conducted using an Axon™ Digidata® 1550B digitizer and an Axopatch 200B amplifier (Molecular Devices), and data were acquired using Clampex 11.1 software.

<u>Translocation experiments</u>

Individual pores were isolated, and their orientation was determined based on the I/V curve (1 M KCl, 15 mM HEPES, pH 7.5). To introduce 2 M urea into the system, 125 µL of the buffer was exchanged with 125 µL of 1 M KCl, 8 M urea, 15 mM HEPES, pH 7.5 in both compartments. Subsequently, 300 nM malE219a was added to the cis compartment, and translocation was induced by applying a negative potential. Each mutant nanopore was tested in triplicate.

<u>Data analysis</u>

The data files generated were analysed using Clampfit 11.1 software. Initially, the open pore current (level 0, L0) and its corresponding noise, σ, were derived from a full point histogram. Subsequently, L1, denoting the detection limit, was defined at 10σ (noting that Clampfit sets its midway detection algorithm at 5σ). Events were captured utilising voltage protocols where the potential was ramped between positive and negative applied potentials. For cis-to-trans translocation events, detection typically occurred at negative potentials within approximately 2 seconds of recording time under negative bias.

The resulting L1 data points were used to create a scatter plot of log(dwell time) versus amplitude, which enabled the determination of amplitude boundaries for the event clusters. Events with durations shorter than 100 µs were disregarded. Subsequently, using these amplitude boundaries, logarithmic histograms of dwell time and conventional histograms of either amplitude or $I_{ex\%}$ were generated. Bin values were set to approximate a Gaussian distribution within the histograms.

Values for log(dwell time) and either amplitude or $I_{ex\%}$ were determined by fitting a Gaussian function to the histogram, with µ representing either log(dwell time) or amplitude/$I_{ex\%}$. Dwell time and $I_{ex\%}$ were reported from triplicate measurements, with values representing the average obtained from three individual repeats alongside the corresponding standard deviation (SD).

**Tables dwell time, velocity and excluded current CytK mutants + malE219a**

| 4D | | | | | | |
|---|---|---|---|---|---|---|
| Voltage (mV) | <t> (ms) | <Iex> (%) | <v> (AA/ms) | SD t | SD Iex | SD v |
| -60 | 24.44 | 6.73 | 17.66 | 1.24 | 72.29 | 3.66 |
| -80 | 10.92 | 0.65 | 37.84 | 0.80 | 71.86 | 2.34 |
| -100 | 5.51 | 0.45 | 75.16 | 2.08 | 71.27 | 6.12 |
| -120 | 3.28 | 0.45 | 127.07 | 5.51 | 70.06 | 16.20 |

| Q5A | | | | | | |
|---|---|---|---|---|---|---|
| Voltage (mV) | <t> (ms) | <Iex> (%) | <v> (AA/ms) | SD t | SD Iex | SD v |
| -60 | 42.96 | 73.80 | 9.72 | 6.35 | 0.74 | 1.33 |
| -80 | 13.61 | 72.75 | 30.58 | 1.76 | 0.36 | 3.71 |
| -100 | 6.93 | 72.30 | 59.47 | 0.15 | 0.86 | 1.28 |
| -120 | 4.86 | 71.58 | 85.61 | 0.64 | 0.10 | 10.47 |

| Q5W | | | | | | |
|---|---|---|---|---|---|---|
| Voltage (mV) | <t> (ms) | <Iex> (%) | <v> (AA/ms) | SD t | SD Iex | SD v |
| -60 | 108.66 | 78.60 | 3.81 | 9.35 | 1.25 | 0.32 |
| -80 | 33.07 | 77.62 | 12.66 | 4.93 | 0.83 | 2.06 |
| -100 | 14.59 | 78.72 | 28.68 | 2.30 | 0.61 | 4.20 |
| -120 | 9.33 | 78.42 | 45.01 | 1.65 | 0.97 | 7.32 |

| Q5Y | | | | | | |
|---|---|---|---|---|---|---|
| Voltage (mV) | <t> (ms) | <Iex> (%) | <v> (AA/ms) | SD t | SD Iex | SD v |
| -60 | 48.17 | 74.94 | 8.78 | 9.60 | 0.11 | 1.75 |
| -80 | 19.51 | 72.98 | 21.14 | 0.78 | 0.61 | 0.86 |
| -100 | 10.55 | 73.24 | 39.08 | 0.21 | 0.87 | 0.80 |
| -120 | 7.01 | 72.42 | 58.87 | 0.33 | 0.69 | 2.82 |
| -140 | 5.38 | 71.99 | 76.92 | 0.48 | 0.62 | 6.67 |
| -160 | 4.00 | 70.34 | 104.54 | 0.56 | 0.24 | 15.41 |

| Q5F | | | | | | |
|---|---|---|---|---|---|---|
| Voltage (mV) | <t> (ms) | <Iex> (%) | <v> (AA/ms) | SD t | SD Iex | SD v |
| -60 | 63.94 | 74.30 | 6.56 | 9.96 | 0.85 | 1.12 |
| -80 | 19.49 | 74.14 | 21.16 | 0.76 | 0.32 | 0.84 |
| -100 | 11.58 | 73.82 | 35.63 | 0.49 | 1.09 | 1.53 |
| -120 | 7.37 | 72.25 | 55.94 | 0.25 | 0.32 | 1.86 |

| Q5E | | | | | | |
|---|---|---|---|---|---|---|
| Voltage (mV) | <t> (ms) | <Iex> (%) | <v> (AA/ms) | SD t | SD Iex | SD v |
| -60 | 44.53 | 74.45 | 9.70 | 10.84 | 0.22 | 3.46 |
| -80 | 11.16 | 73.49 | 37.14 | 1.07 | 0.26 | 3.54 |
| -100 | 7.07 | 72.26 | 58.37 | 0.32 | 0.18 | 2.71 |
| -120 | 4.20 | 71.52 | 98.28 | 0.17 | 0.25 | 4.10 |

| Q5R | | | | | | |
|---|---|---|---|---|---|---|
| Voltage (mV) | <t> (ms) | <Iex> (%) | <v> (AA/ms) | SD t | SD Iex | SD v |
| -60 | 57.19 | 72.58 | 7.32 | 9.32 | 0.36 | 1.10 |
| -80 | 18.50 | 73.05 | 22.77 | 3.37 | 1.82 | 4.10 |
| -100 | 10.57 | 71.37 | 40.56 | 2.72 | 1.38 | 9.11 |
| -120 | 6.57 | 69.73 | 63.06 | 0.58 | 1.07 | 5.70 |

| T114F | | | | | | |
|---|---|---|---|---|---|---|
| Voltage (mV) | <t> (ms) | <Iex> (%) | <v> (AA/ms) | SD t | SD Iex | SD v |
| -60 | 51.98 | 78.61 | 7.97 | 4.55 | 0.15 | 0.71 |
| -80 | 16.92 | 79.34 | 24.36 | 0.34 | 0.52 | 0.49 |
| -100 | 11.55 | 78.91 | 35.67 | 0.20 | 0.16 | 0.62 |
| -120 | 7.34 | 79.09 | 56.26 | 0.47 | 1.77 | 3.63 |

| S126F | | | | | | |
|---|---|---|---|---|---|---|
| Voltage (mV) | <t> (ms) | <Iex> (%) | <v> (AA/ms) | SD t | SD Iex | SD v |
| -60 | 210.73 | 88.27 | 1.96 | 14.77 | 0.17 | 0.14 |
| -80 | 83.70 | 88.67 | 4.94 | 6.40 | 0.26 | 0.38 |
| -100 | 52.32 | 89.23 | 8.01 | 2.60 | 0.63 | 0.33 |
| -120 | 40.17 | 90.53 | 10.32 | 4.63 | 0.00 | 1.19 |
| -140 | 29.01 | 90.71 | 14.20 | 0.24 | 0.51 | 0.12 |

| S126I | | | | | | |
|---|---|---|---|---|---|---|
| Voltage (mV) | <t> (ms) | <Iex> (%) | <v> (AA/ms) | SD t | SD Iex | SD v |
| -60 | 143.11 | 83.16 | 2.91 | 19.30 | 0.74 | 0.40 |
| -80 | 50.86 | 83.93 | 8.11 | 2.47 | 0.67 | 0.38 |
| -100 | 31.33 | 84.12 | 13.15 | 0.40 | 0.32 | 0.17 |
| -120 | 23.02 | 83.90 | 17.94 | 1.37 | 0.49 | 1.10 |
| -140 | 14.29 | 83.31 | 28.54 | 0.21 | 0.71 | 0.42 |

| T143F | | | | | | |
|---|---|---|---|---|---|---|

| Voltage (mV) | <t> (ms) | <Iex> (%) | <v> (AA/ms) | SD t | SD Iex | SD v |
|---|---|---|---|---|---|---|
| -60 | 162.26 | 86.07 | 2.56 | 18.55 | 0.92 | 0.29 |
| -80 | 79.10 | 87.40 | 5.28 | 10.76 | 0.29 | 0.78 |
| -100 | 53.49 | 87.89 | 7.74 | 4.37 | 0.79 | 0.61 |
| -120 | 35.88 | 88.34 | 11.73 | 6.21 | 0.36 | 2.17 |

| T147F | | | | | | |
|---|---|---|---|---|---|---|
| Voltage (mV) | <t> (ms) | <Iex> (%) | <v> (AA/ms) | SD t | SD Iex | SD v |
| -60 | 178.40 | 85.33 | 2.37 | 36.29 | 0.28 | 0.45 |
| -80 | 59.66 | 85.23 | 7.03 | 10.16 | 0.65 | 1.10 |
| -100 | 32.22 | 86.14 | 12.85 | 2.86 | 0.28 | 1.09 |
| -120 | 19.78 | 86.90 | 21.01 | 2.27 | 0.30 | 2.32 |

| T147I | | | | | | |
|---|---|---|---|---|---|---|
| Voltage (mV) | <t> (ms) | <Iex> (%) | <v> (AA/ms) | SD t | SD Iex | SD v |
| -60 | 88.03 | 78.57 | 4.81 | 16.68 | 1.39 | 1.01 |
| -80 | 27.59 | 78.70 | 15.28 | 5.25 | 0.83 | 2.81 |
| -100 | 14.41 | 77.53 | 29.03 | 2.27 | 0.96 | 4.27 |
| -120 | 8.04 | 78.19 | 51.52 | 0.70 | 0.41 | 4.43 |
| -140 | 5.75 | 76.91 | 71.87 | 0.40 | 1.10 | 4.87 |

| S149F | | | | | | |
|---|---|---|---|---|---|---|
| Voltage (mV) | <t> (ms) | <Iex> (%) | <v> (AA/ms) | SD t | SD Iex | SD v |
| -60 | 193.63 | 83.35 | 2.18 | 37.74 | 0.67 | 0.44 |
| -80 | 35.62 | 82.29 | 11.57 | 0.85 | 0.54 | 0.28 |
| -100 | 18.55 | 83.04 | 22.22 | 0.45 | 0.10 | 0.54 |
| -120 | 12.64 | 81.36 | 33.51 | 2.62 | 1.47 | 6.66 |

| S149I | | | | | | |
|---|---|---|---|---|---|---|
| Voltage (mV) | <t> (ms) | <Iex> (%) | <v> (AA/ms) | SD t | SD Iex | SD v |
| -60 | 103.87 | 77.39 | 4.15 | 25.01 | 0.71 | 1.16 |
| -80 | 28.90 | 77.03 | 14.36 | 3.02 | 0.71 | 1.45 |
| -100 | 15.70 | 77.07 | 26.24 | 0.10 | 0.08 | 0.16 |
| -120 | 9.06 | 76.57 | 45.63 | 0.62 | 0.30 | 3.01 |
| -140 | 6.33 | 75.73 | 65.28 | 0.39 | 0.24 | 3.96 |

| Q5Y-S149I | | | | | | |
|---|---|---|---|---|---|---|
| Voltage (mV) | <t> (ms) | <Iex> (%) | <v> (AA/ms) | SD t | SD Iex | SD v |
| -60 | 212.07 | 79.56 | 1.94 | 6.73 | 0.36 | 0.06 |
| -80 | 49.30 | 79.14 | 8.49 | 7.69 | 0.77 | 1.33 |
| -100 | 21.55 | 79.17 | 19.18 | 1.51 | 0.19 | 1.29 |
| -120 | 13.27 | 78.95 | 31.21 | 1.20 | 0.57 | 2.72 |
| -140 | 9.41 | 78.19 | 44.18 | 1.13 | 0.58 | 5.20 |

| T114F-S126F | | | | | | |
|---|---|---|---|---|---|---|
| Voltage (mV) | <t> (ms) | <Iex> (%) | <v> (AA/ms) | SD t | SD Iex | SD v |
| -60 | 278.70 | 88.93 | 1.48 | 21.00 | 0.24 | 0.11 |
| -80 | 132.59 | 90.08 | 3.11 | 4.71 | 0.16 | 0.11 |
| -100 | 88.96 | 91.67 | 4.64 | 3.37 | 0.29 | 0.18 |
| -120 | 51.84 | 92.77 | 7.96 | 2.60 | 0.96 | 0.39 |

| Q5Y-T114F | | | | | | |
|---|---|---|---|---|---|---|
| Voltage (mV) | <t> (ms) | <Iex> (%) | <v> (AA/ms) | SD t | SD Iex | SD v |
| -60 | 66.33 | 78.48 | 6.22 | 3.54 | 0.19 | 0.33 |
| -80 | 29.12 | 79.22 | 14.34 | 3.84 | 0.53 | 1.97 |
| -100 | 18.09 | 79.70 | 23.44 | 3.51 | 0.39 | 4.61 |
| -120 | 14.29 | 81.65 | 28.84 | 0.42 | 2.99 | 0.87 |

| Q5Y-T114F-S126F | | | | | | |
|---|---|---|---|---|---|---|
| Voltage (mV) | <t> (ms) | <Iex> (%) | <v> (AA/ms) | SD t | SD Iex | SD v |
| -60 | 351.65 | 89.27 | 1.20 | 67.47 | 0.57 | 0.21 |
| -80 | 152.78 | 89.85 | 2.70 | 3.33 | 0.39 | 0.06 |
| -100 | 92.87 | 91.83 | 4.45 | 5.83 | 0.58 | 0.27 |
| -120 | 82.28 | 93.07 | 5.05 | 9.72 | 0.36 | 0.57 |
| -140 | 74.31 | 94.58 | 5.58 | 7.13 | 0.66 | 0.55 |
| -160 | 82.99 | 95.85 | 4.96 | | | |

**Tables dwell time, velocity and excluded current CytK mutants + tzatziki**

| CytK-4D + tzatziki | | | | | | |
|---|---|---|---|---|---|---|
| Voltage (mV) | <t> (ms) | <Iex> (%) | <v> (AA/ms) | SD t | SD Iex | SD v |
| -100 | 5.74 | 92.83 | 28.86 | 2.39 | 0.25 | 14.82 |
| -120 | 1.74 | 91.37 | 80.84 | 0.18 | 1.95 | 7.64 |
| -140 | 1.05 | 89.93 | 134.17 | 0.10 | 2.07 | 12.97 |
| -160 | 0.66 | 88.79 | 213.77 | 0.06 | 2.03 | 19.80 |

| | | | | | | |
|---|---|---|---|---|---|---|
| -180 | 0.42 | 87.02 | 337.66 | 0.05 | 2.55 | 42.48 |

| Q5Y + tzatziki | | | | | | |
|---|---|---|---|---|---|---|
| Voltage (mV) | <t> (ms) | <Iex> (%) | <v> (AA/ms) | SD t | SD Iex | SD v |
| -100 | 7.86 | 92.21 | 17.94 | 0.81 | 0.31 | 1.91 |
| -120 | 3.19 | 92.06 | 47.37 | 1.10 | 0.17 | 15.28 |
| -140 | 1.38 | 91.26 | 103.74 | 0.24 | 0.08 | 19.15 |
| -160 | 0.90 | 90.34 | 157.42 | 0.09 | 0.19 | 16.43 |
| -180 | 0.61 | 89.35 | 234.16 | 0.10 | 0.15 | 43.08 |

| T114F + tzatziki | | | | | | |
|---|---|---|---|---|---|---|
| Voltage (mV) | <t> (ms) | <Iex> (%) | <v> (AA/ms) | SD t | SD Iex | SD v |
| -100 | 13.11 | 93.27 | 11.31 | 4.09 | 0.16 | 3.06 |
| -120 | 3.52 | 93.27 | 40.07 | 0.34 | 0.32 | 3.90 |
| -140 | 1.64 | 92.12 | 85.66 | 0.08 | 0.20 | 3.93 |
| -160 | 0.90 | 91.34 | 156.21 | 0.04 | 0.32 | 6.52 |
| -180 | 0.70 | 90.39 | 200.14 | 0.04 | 0.14 | 12.40 |

| S126F + tzatziki | | | | | | |
|---|---|---|---|---|---|---|
| Voltage (mV) | <t> (ms) | <Iex> (%) | <v> (AA/ms) | SD t | SD Iex | SD v |
| -100 | 18.17 | 96.45 | 9.93 | 12.37 | 0.56 | 4.91 |
| -120 | 4.03 | 95.45 | 35.39 | 0.72 | 0.63 | 5.75 |
| -140 | 1.64 | 93.86 | 86.96 | 0.25 | 0.16 | 14.54 |
| -160 | 1.03 | 92.61 | 137.14 | 0.09 | 0.12 | 11.38 |
| -180 | 0.77 | 91.68 | 181.64 | 0.07 | 0.04 | 15.77 |

| S126F + tzatziki | | | | | | |
|---|---|---|---|---|---|---|
| Voltage (mV) | <t> (ms) | <Iex> (%) | <v> (AA/ms) | SD t | SD Iex | SD v |
| -100 | 17.63 | 96.38 | 8.88 | 7.62 | 0.17 | 3.32 |
| -120 | 5.85 | 95.59 | 26.31 | 2.19 | 0.19 | 9.78 |
| -140 | 2.64 | 94.80 | 53.49 | 0.26 | 0.38 | 5.54 |
| -160 | 1.65 | 93.85 | 87.39 | 0.33 | 0.23 | 17.79 |
| -180 | 1.10 | 93.16 | 131.59 | 0.23 | 0.39 | 30.64 |

**Tables dwell time, velocity and excluded current CytK Q5Y-S149I + GBP or DHFR proteins**

| Q5Y-S149I + GBP H152A | | | | | | |
|---|---|---|---|---|---|---|
| Voltage (mV) | <t> (ms) | <Iex> (%) | <v> (AA/ms) | SD t | SD Iex | SD v |
| -80 | 8.34 | 78.03 | 38.24 | 0.05 | 0.42 | 0.22 |
| -100 | 6.04 | 77.12 | 52.94 | 0.39 | 0.60 | 3.52 |
| -120 | 3.88 | 77.33 | 83.65 | 0.62 | 0.66 | 14.16 |
| -140 | 2.92 | 76.86 | 109.34 | 0.13 | 0.76 | 5.18 |
| -160 | 2.02 | 77.72 | 159.14 | 0.24 | 0.39 | 18.60 |

| Q5Y-S149I + DHFR W30G W133L | | | | | | |
|---|---|---|---|---|---|---|
| Voltage (mV) | <t> (ms) | <Iex> (%) | <v> (AA/ms) | SD t | SD Iex | SD v |
| -80 | 27.16 | 80.24 | 6.35 | 4.12 | 0.11 | 0.95 |
| -100 | 15.35 | 76.92 | 11.46 | 3.45 | 1.51 | 2.57 |
| -120 | 7.90 | 74.74 | 21.78 | 1.03 | 0.61 | 2.91 |
| -140 | 4.45 | 72.10 | 38.69 | 0.60 | 0.94 | 5.10 |
| -160 | 3.02 | 70.51 | 56.49 | 0.23 | 0.49 | 4.44 |

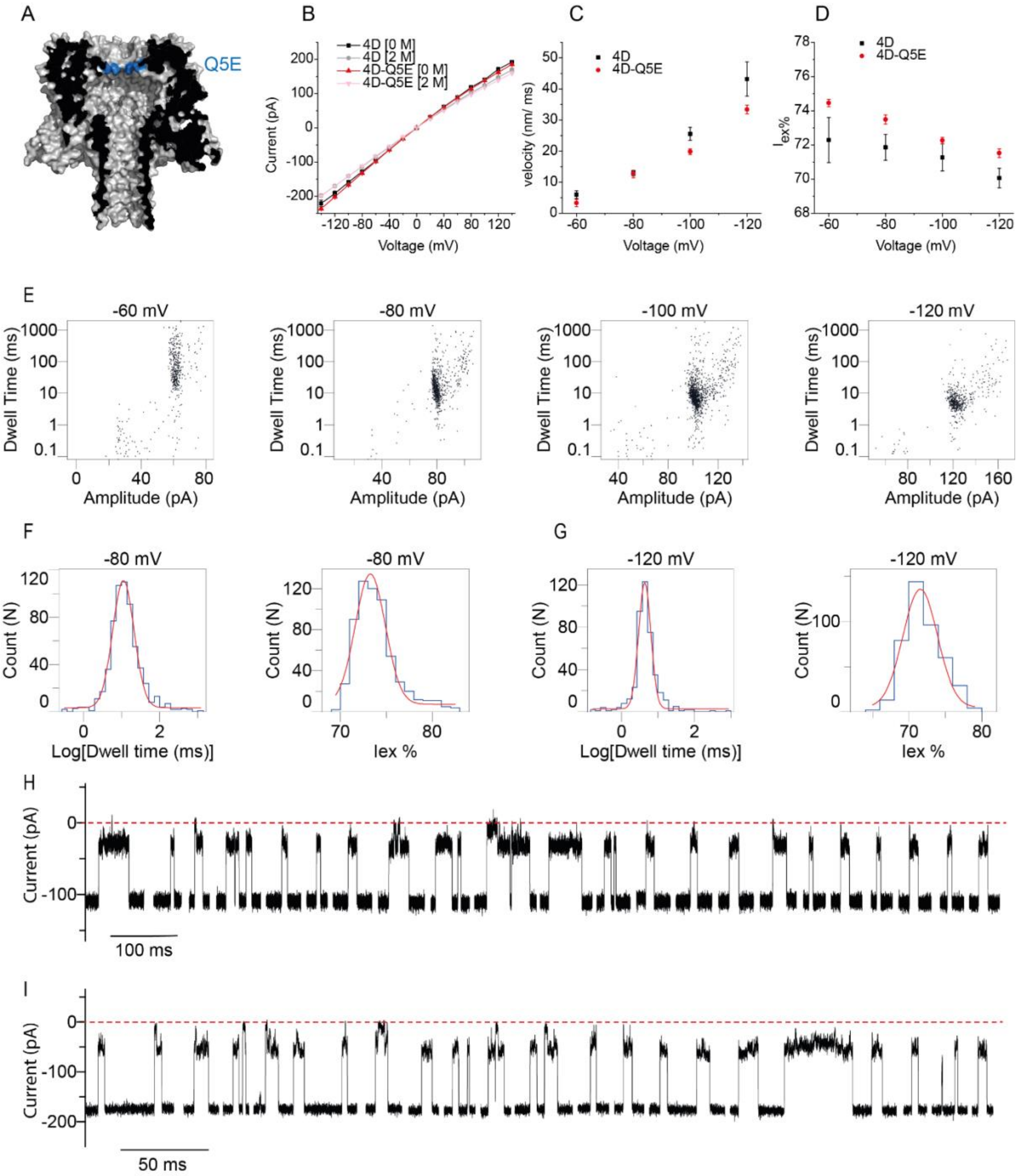


***Figure S1. Translocation of malE219a through the CytK 4D-Q5E. A**) The CytK nanopore, where the Q5 position is highlighted. **B)** IV curves in 1 M KCl at pH 7.5 of the 4D and 4D-Q5E mutants in 0 and 2 M urea; each curve was obtained from a triplicate measurement. **C)** Translocation velocity dependence of malE219a through the 4D and 4D-Q5E. **D)** $I_{ex\%}$ dependence of malE219a translocation through the 4D and 4D-Q5E. Data points represent averages from three independent experiments and the error bars correspond to standard deviations (SD). **E)** Scatter plots (dwell time vs amplitude) associated with the malE219a translocation at the sampled potentials. **F-G)** Examples of histograms obtained for the log(dwell time) and Iex at -80 mV and -120 mV, respectively. **H-I)** Typical malE219a translocation events at -80 mV (H) and -120 mV (I). Recordings were carried out in 1 M KCl, 15 mM HEPES, 2 M urea, pH 7.5, 50 kHz sampling and 10 kHz Bessel filter.*

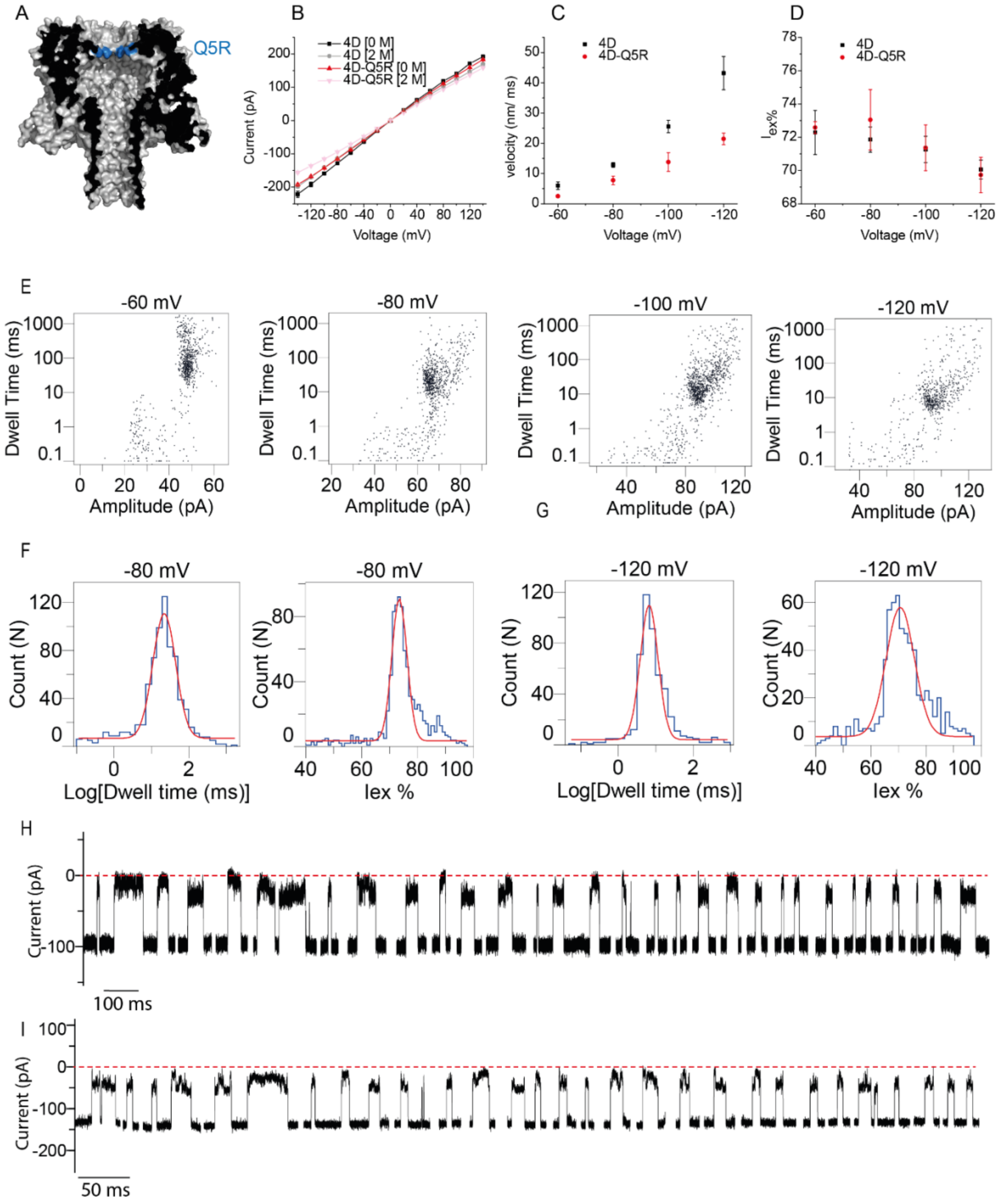


***Figure S2. Translocation of malE219a through the CytK 4D-Q5R. A****) The CytK nanopore, where the Q5 position is highlighted.* ***B)*** *IV curves in 1 M KCl at pH 7.5 of the 4D and 4D-Q5R mutants in 0 and 2 M urea; each curve was obtained from a triplicate measurement.* ***C)*** *Translocation velocity dependence of malE219a through the 4D and 4D-Q5R.* ***D)*** *$I_{ex\%}$ dependence of malE219a translocation through the 4D and 4D-Q5R. Data points represent averages from three independent experiments and the error bars correspond to standard deviations (SD).* ***E)*** *Scatter plots (dwell time vs amplitude) associated with the malE219a translocation at the sampled potentials.* ***F-G)*** *Examples of histograms obtained for the log(dwell time) and Iex at -80 mV and -120 mV, respectively.* ***H-I)*** *Typical malE219a translocation events at -80 mV (H) and -120 mV (I). Recordings were carried out in 1 M KCl, 15 mM HEPES, 2 M urea, pH 7.5, 50 kHz sampling and 10 kHz Bessel filter.*

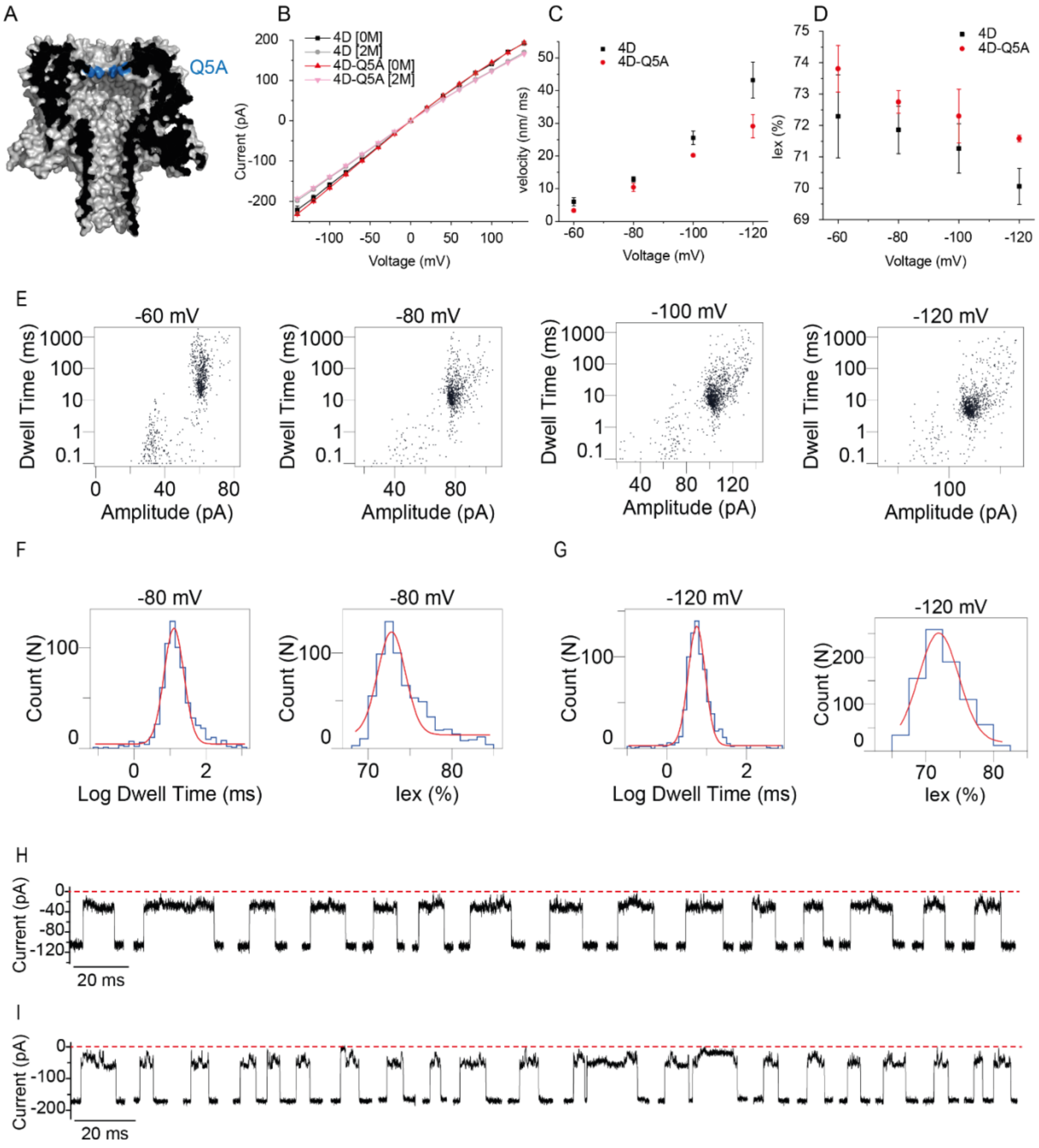


***Figure S3*. *Translocation of malE219a through the CytK 4D-Q5A. A*)** *The CytK nanopore, where the Q5 position is highlighted.* ***B)*** *IV curves in 1 M KCl at pH 7.5 of the 4D and 4D-Q5A mutants in 0 and 2 M urea; each curve was obtained from a triplicate measurement.* ***C)*** *Translocation velocity dependence of malE219a through the 4D and 4D-Q5A.* ***D)*** *$I_{ex\%}$ dependence of malE219a translocation through the 4D and 4D-Q5A. Data points represent averages from three independent experiments and the error bars correspond to standard deviations (SD).* ***E)*** *Scatter plots (dwell time vs amplitude) associated with the malE219a translocation at the sampled potentials.* ***F-G)*** *Examples of histograms obtained for the log(dwell time) and Iex at -80 mV and -120 mV, respectively.* ***H-I)*** *Typical malE219a translocation events at -80 mV (H) and -120 mV (I). Recordings were carried out in 1 M KCl, 15 mM HEPES, 2 M urea, pH 7.5, 50 kHz sampling and 10 kHz Bessel filter.*

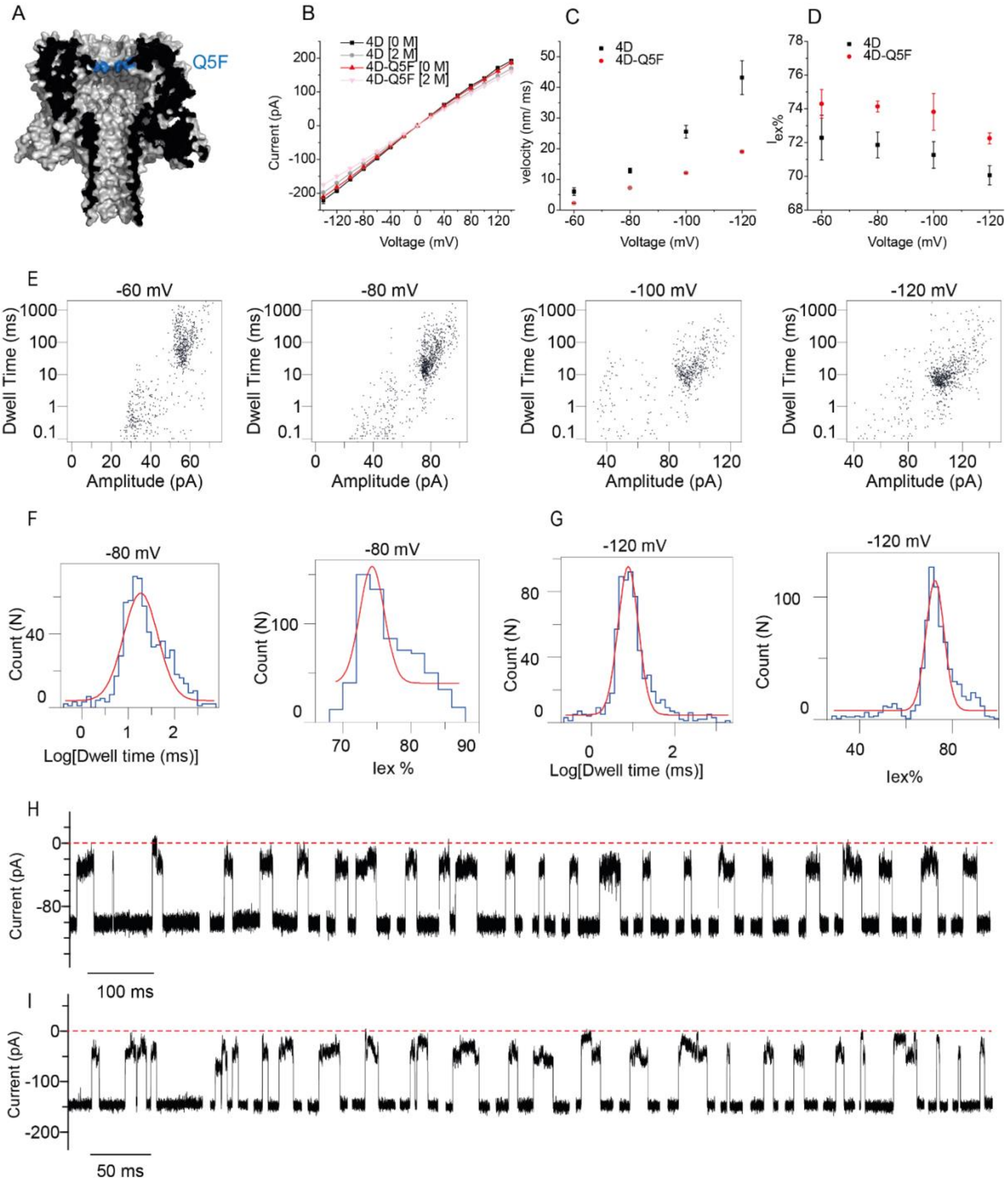


***Figure S4. Translocation of malE219a through the CytK 4D-Q5F. A**) The CytK nanopore, where the Q5 position is highlighted.* ***B)*** *IV curves in 1 M KCl at pH 7.5 of the 4D and 4D-Q5F mutants in 0 and 2 M urea; each curve was obtained from a triplicate measurement.* ***C)*** *Translocation velocity dependence of malE219a through the 4D and 4D-Q5F.* ***D)*** *$I_{ex\%}$ dependence of malE219a translocation through the 4D and 4D-Q5F. Data points represent averages from three independent experiments and the error bars correspond to standard deviations (SD).* ***E)*** *Scatter plots (dwell time vs amplitude) associated with the malE219a translocation at the sampled potentials.* ***F-G)*** *Examples of histograms obtained for the log(dwell time) and Iex at -80 mV and -120 mV, respectively.* ***H-I)*** *Typical malE219a translocation events at -80 mV (H) and -120 mV (I). Recordings were carried out in 1 M KCl, 15 mM HEPES, 2 M urea, pH 7.5, 50 kHz sampling and 10 kHz Bessel filter.*

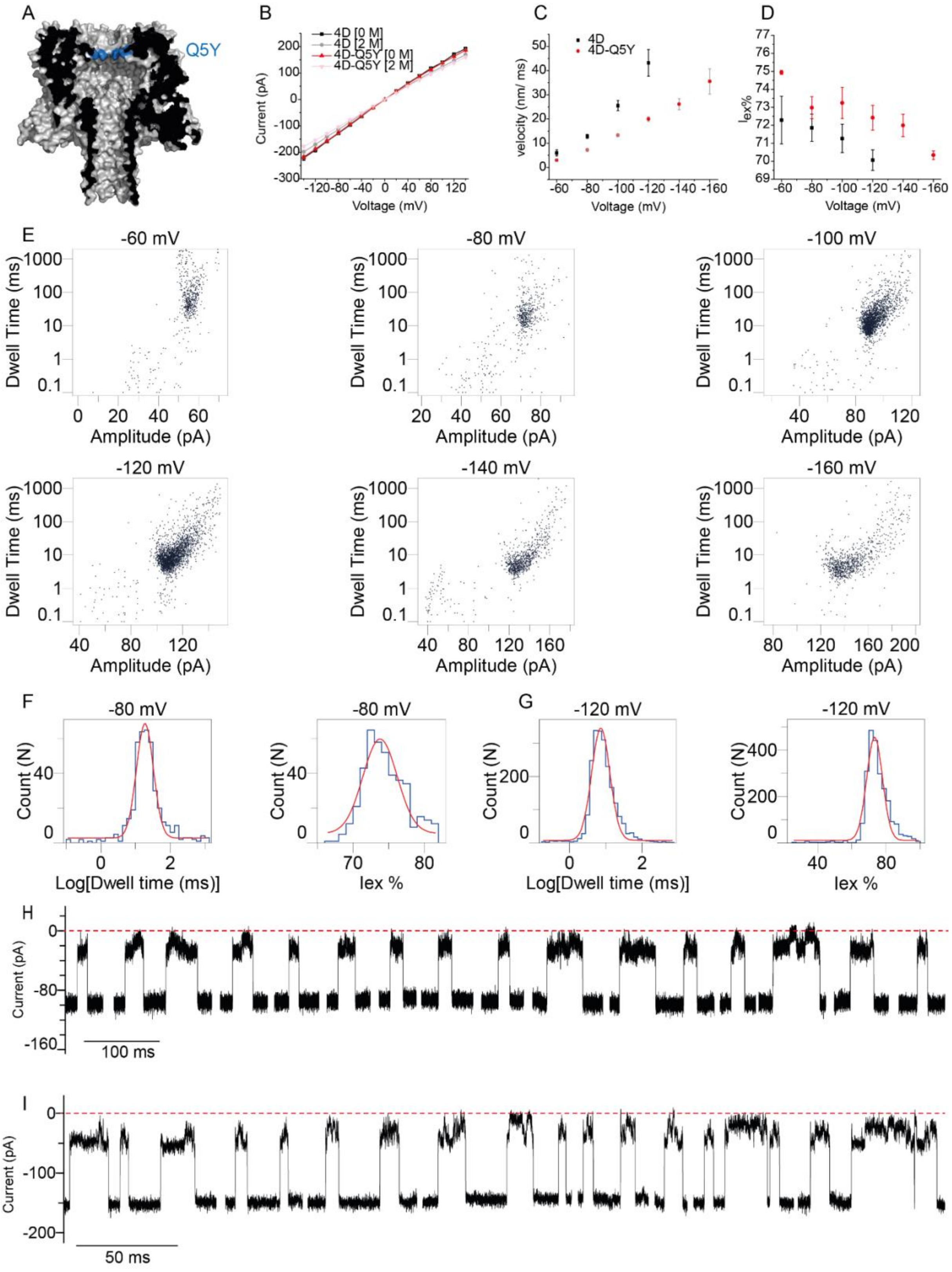

***Figure S5. Translocation of malE219a through the CytK 4D-Q5Y. A)*** *The CytK nanopore, where the Q5 position is highlighted.* ***B)*** *IV curves in 1 M KCl at pH 7.5 of the 4D and 4D-Q5Y mutants in 0 and 2 M urea; each curve was obtained from a triplicate measurement.* ***C)*** *Translocation velocity dependence of malE219a through the 4D and 4D-Q5Y.* ***D)*** *$I_{ex\%}$ dependence of malE219a translocation through the 4D and 4D-Q5Y. Data points represent averages from three independent experiments and the error bars correspond to standard deviations (SD).* ***E)*** *Scatter plots (dwell time vs amplitude) associated with the malE219a translocation at the sampled potentials.* ***F-***

***G)*** *Examples of histograms obtained for the log(dwell time) and Iex at -80 mV and -120 mV, respectively.* ***H-I)*** *Typical malE219a translocation events at -80 mV (H) and -120 mV (I). Recordings were carried out in 1 M KCl, 15 mM HEPES, 2 M urea, pH 7.5, 50 kHz sampling and 10 kHz Bessel filter.*

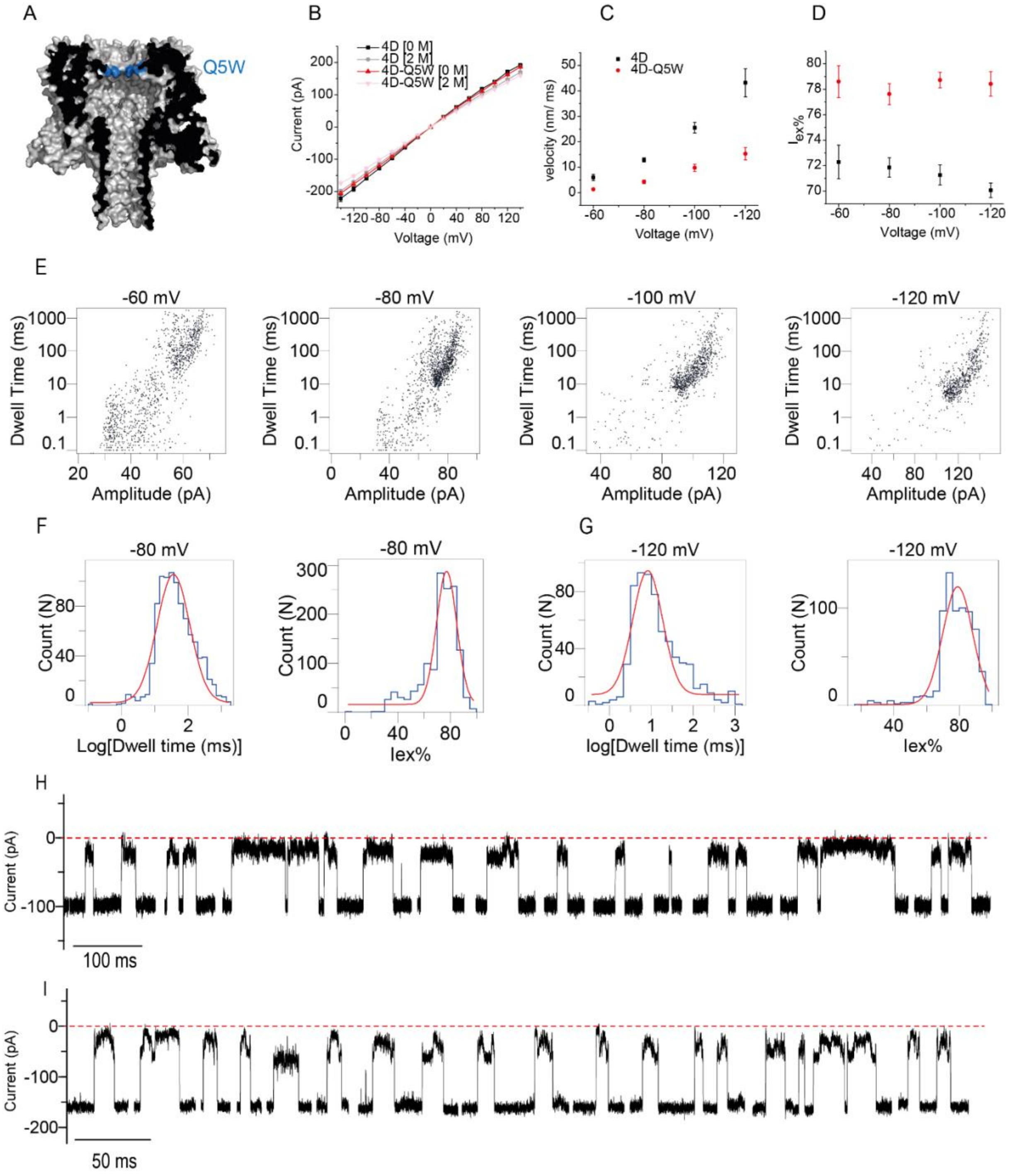

***Figure S6. Translocation of malE219a through the CytK 4D-Q5W. A)*** *The CytK nanopore, where the Q5 position is highlighted.* ***B)*** *IV curves in 1 M KCl at pH 7.5 of the 4D and 4D-Q5W mutants in 0 and 2 M urea; each curve was obtained from a triplicate measurement.* ***C)*** *Translocation velocity dependence of malE219a through the 4D and 4D-Q5W.* ***D)*** *$I_{ex\%}$ dependence of malE219a translocation through the 4D and 4D-Q5W. Data points represent averages from three independent experiments and the error bars correspond to standard deviations (SD).* ***E)*** *Scatter plots (dwell time vs amplitude) associated with the malE219a translocation at the sampled potentials.* ***F-G)*** *Examples of histograms obtained for the log(dwell time) and Iex at -80 mV and -120 mV, respectively.* ***H-I)*** *Typical malE219a translocation events at -80 mV (H) and -120 mV (I). Recordings were carried out in 1 M KCl, 15 mM HEPES, 2 M urea, pH 7.5, 50 kHz sampling and 10 kHz Bessel filter.*

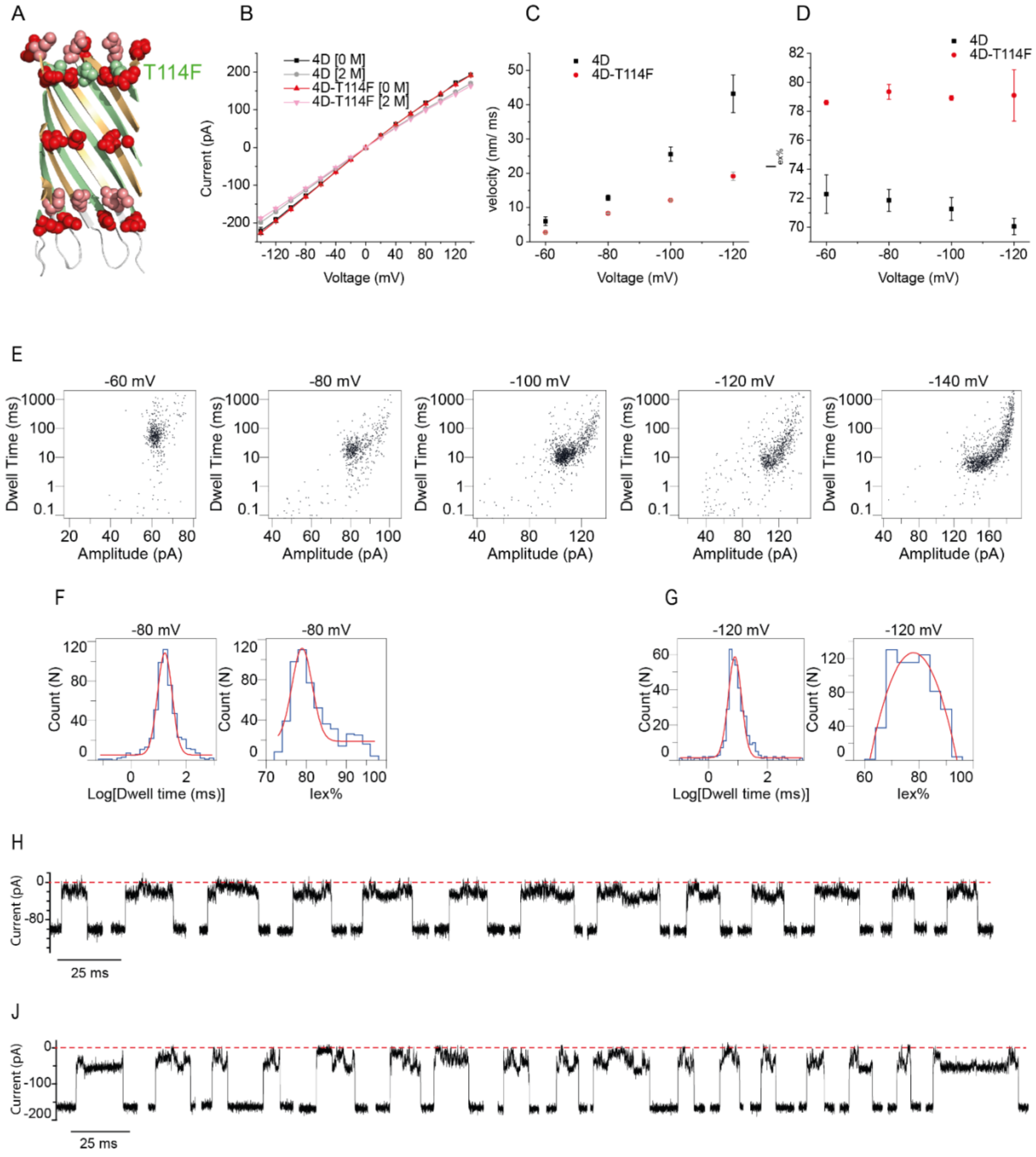

***Figure S7. Translocation of malE219a through the CytK 4D-T114F nanopore.*** ***A)*** *The barrel of the CytK nanopore. The N-term strand is depicted in green, while the C-term strand in green. The relevant residues are shown as spheres: E112 and E139 in pink, the four Asp positions in red (K128, Q145, S151 and K155) and T114 in green.* ***B)*** *IV curves in 1 M KCl at pH 7.5 of the 4D and 4D-T114F mutants in 0 and 2 M urea; each curve was obtained from a triplicate measurement.* ***C)*** *Translocation velocity dependence of malE219a through the 4D and 4D-T114F.* ***D)*** *$I_{ex\%}$ dependence of malE219a translocation through the 4D and 4D-T114F. Data points represent averages from three independent experiments and the error bars correspond to standard deviations (SD).* ***E)*** *Scatter plots (dwell time vs amplitude) associated with the malE219a translocation at the sampled potentials.* ***F-G)*** *Examples of histograms obtained for the log(dwell time) and Iex at -80 mV and -120 mV, respectively.* ***H-I)*** *Typical malE219a translocation events at -80 mV (H) and -120 mV (I). Recordings were carried out in 1 M KCl, 15 mM HEPES, 2 M urea, pH 7.5, 50 kHz sampling and 10 kHz Bessel filter.*

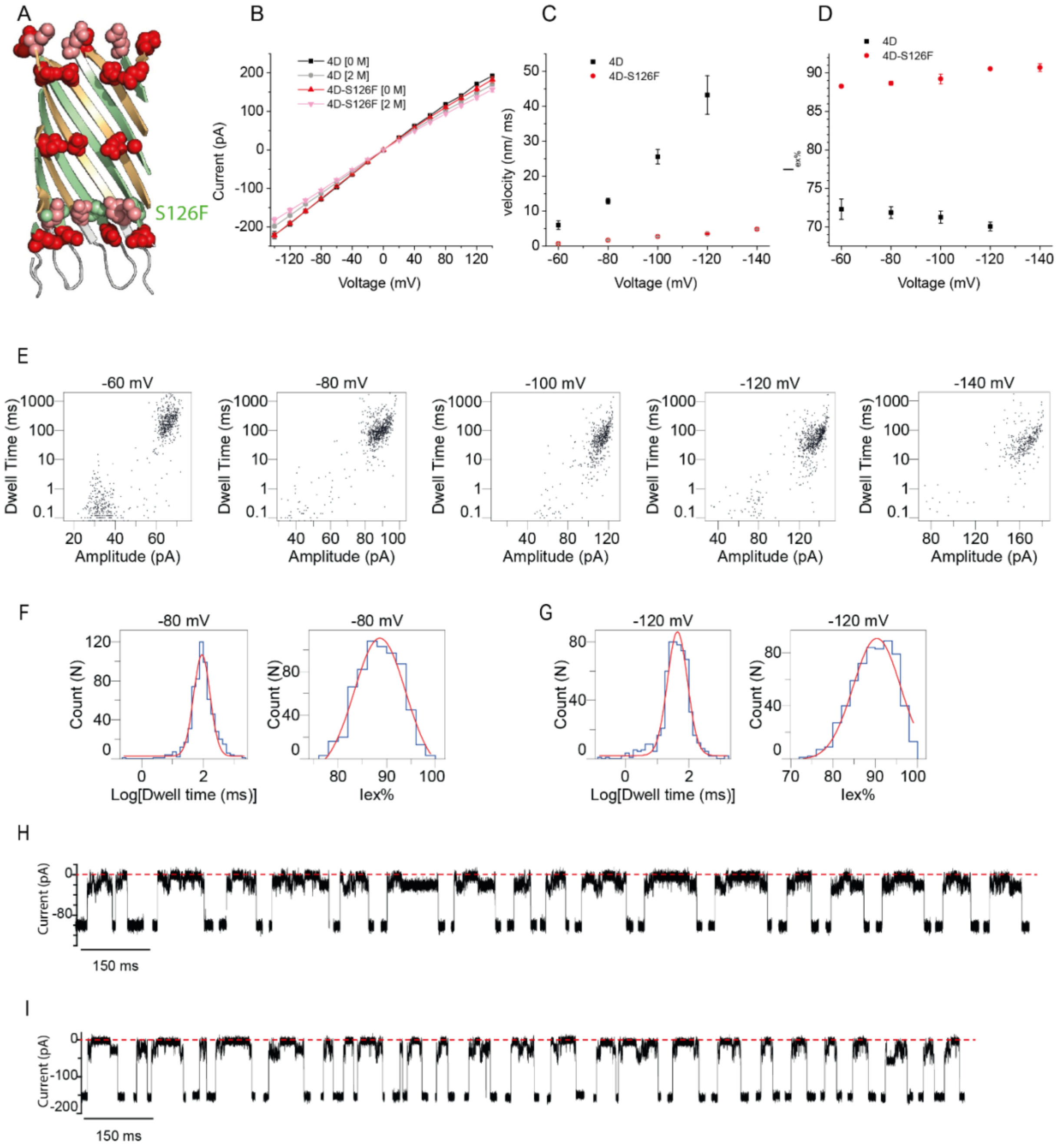


***Figure S8. Translocation of malE219a through the CytK 4D-S126F nanopore.*** ***A)*** *The barrel of the CytK nanopore. The N-term strand is depicted in green, while the C-term strand in green. The relevant residues are shown as spheres: E112 and E139 in pink, the four Asp positions in red (K128, Q145, S151 and K155) and S126 in green.* ***B)*** *IV curves in 1 M KCl at pH 7.5 of the 4D and 4D-S126F mutants in 0 and 2 M urea; each curve was obtained from a triplicate measurement.* ***C)*** *Translocation velocity dependence of malE219a through the 4D and 4D-S126F.* ***D)*** *$I_{ex\%}$ dependence of malE219a translocation through the 4D and 4D-S126F. Data points represent averages from three independent experiments and the error bars correspond to standard deviations (SD).* ***E)*** *Scatter plots (dwell time vs amplitude) associated with the malE219a translocation at the sampled potentials.* ***F-G)*** *Examples of histograms obtained for the log(dwell time) and Iex at -80 mV and -120 mV, respectively.* ***H-I)*** *Typical malE219a translocation events at -80 mV (H) and -120 mV (I). Recordings were carried out in 1 M KCl, 15 mM HEPES, 2 M urea, pH 7.5, 50 kHz sampling and 10 kHz Bessel filter.*

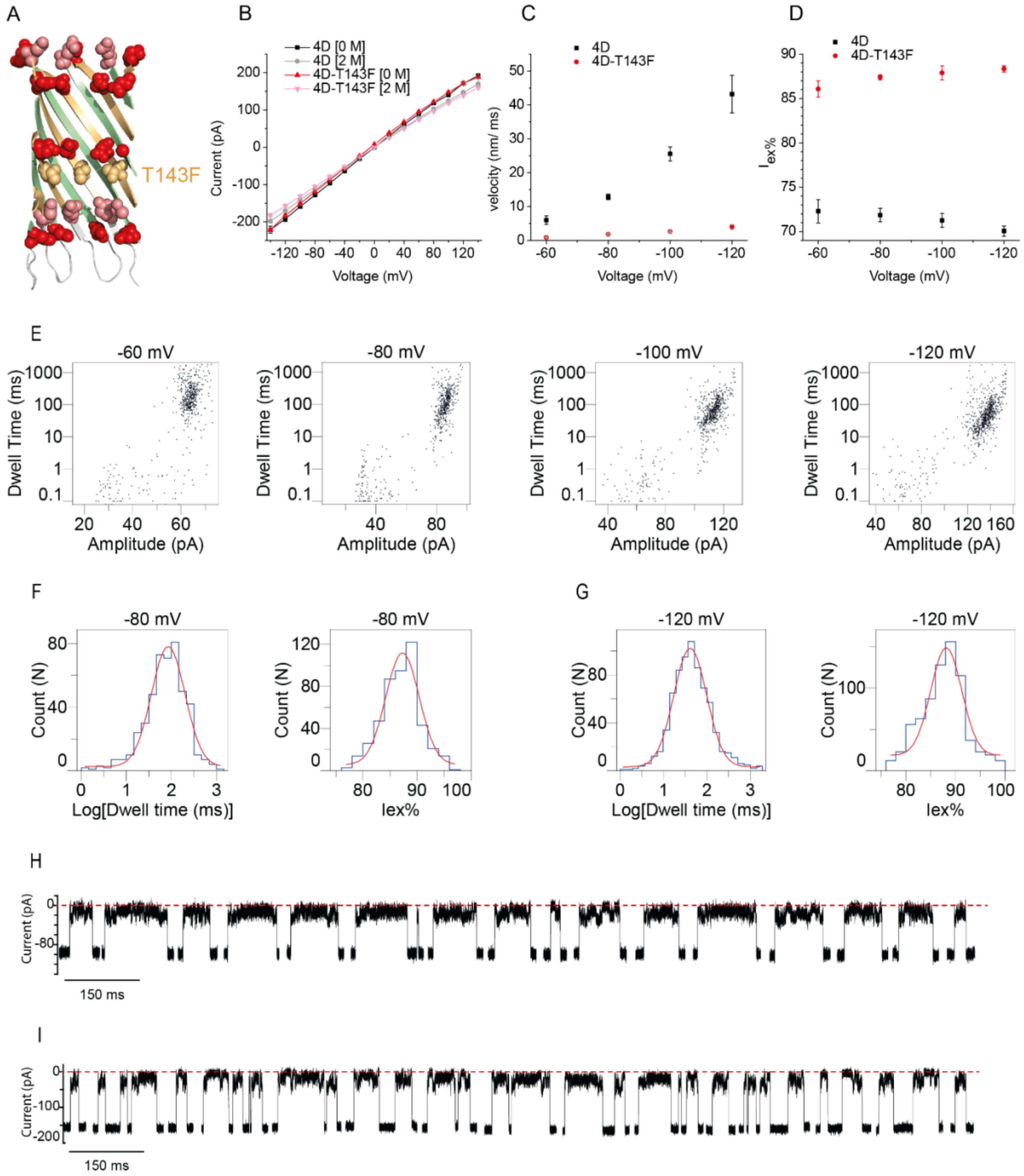


***Figure S9. Translocation of malE219a through the CytK 4D-T143F nanopore. A)*** *The barrel of the CytK nanopore. The N-term strand is depicted in green, while the C-term strand in green. The relevant residues are shown as spheres: E112 and E139 in pink, the four Asp positions in red (K128, Q145, S151 and K155) and T143 in orange.* ***B)*** *IV curves in 1 M KCl at pH 7.5 of the 4D and 4D-T143F mutants in 0 and 2 M urea; each curve was obtained from a triplicate measurement.* ***C)*** *Translocation velocity dependence of malE219a through the 4D and 4D-T143F.* ***D)*** *$I_{ex\%}$ dependence of malE219a translocation through the 4D and 4D-T143F. Data points represent averages from three independent experiments and the error bars correspond to standard deviations (SD).* ***E)*** *Scatter plots (dwell time vs amplitude) associated with the malE219a translocation at the sampled potentials.* ***F-G)*** *Examples of histograms obtained for the log(dwell time) and Iex at -80 mV and -120 mV, respectively.* ***H-I)*** *Typical malE219a translocation events at -80 mV (H) and -120 mV (I). Recordings were carried out in 1 M KCl, 15 mM HEPES, 2 M urea, pH 7.5, 50 kHz sampling and 10 kHz Bessel filter.*

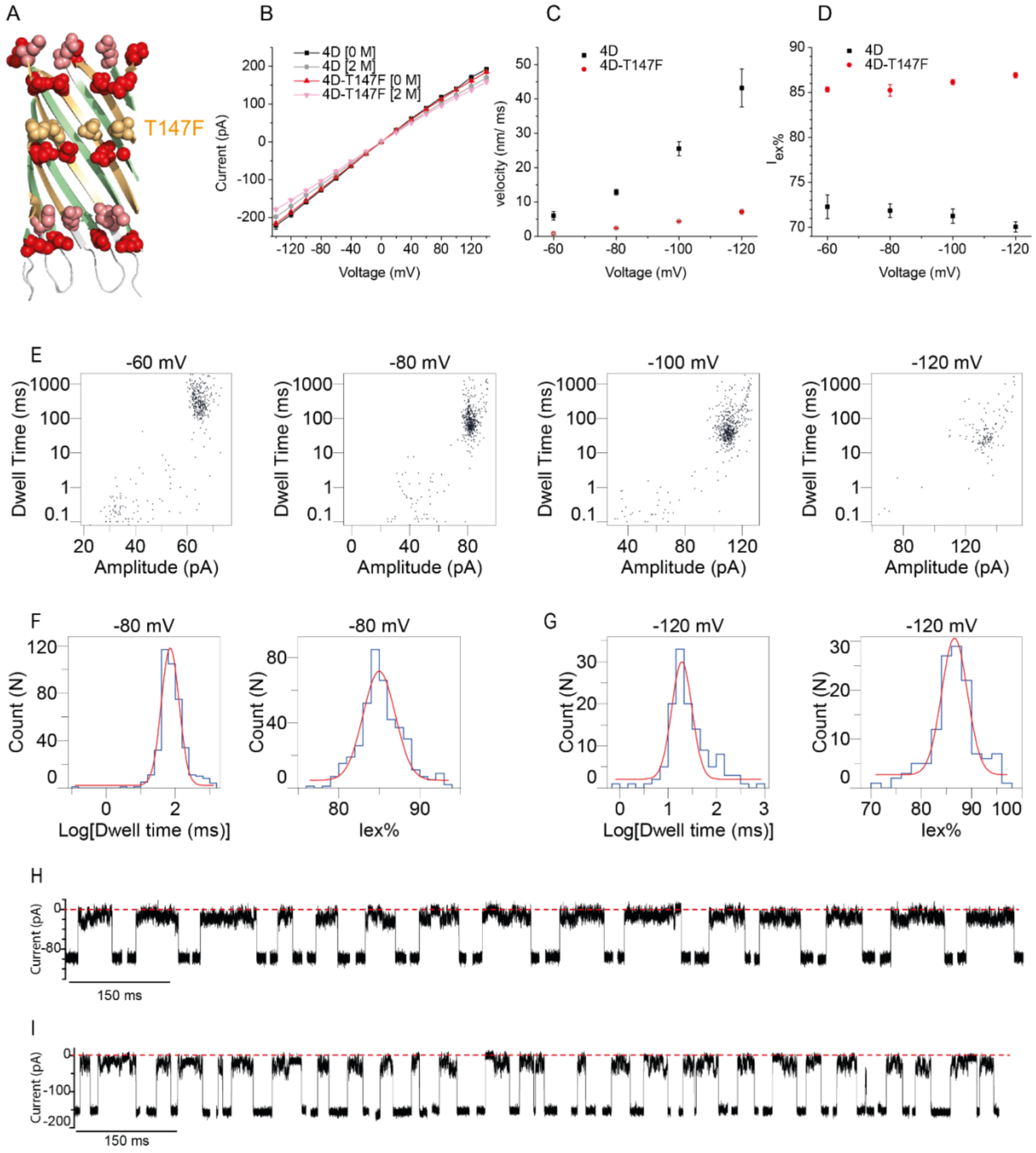


***Figure S10. Translocation of malE219a through the CytK 4D-T147F nanopore. A)*** *The barrel of the CytK nanopore. The N-term strand is depicted in green, while the C-term strand in green. The relevant residues are shown as spheres: E112 and E139 in pink, the four Asp positions in red (K128, Q145, S151 and K155) and T147 in orange.* ***B)*** *IV curves in 1 M KCl at pH 7.5 of the 4D and 4D-T147F mutants in 0 and 2 M urea; each curve was obtained from a triplicate measurement.* ***C)*** *Translocation velocity dependence of malE219a through the 4D and 4D-T147F.* ***D)*** *$I_{ex\%}$ dependence of malE219a translocation through the 4D and 4D-T147F. Data points represent averages from three independent experiments and the error bars correspond to standard deviations (SD).* ***E)*** *Scatter plots (dwell time vs amplitude) associated with the malE219a translocation at the sampled potentials.* ***F-G)*** *Examples of histograms obtained for the log(dwell time) and Iex at -80 mV and -120 mV, respectively.* ***H-I)*** *Typical malE219a translocation events at -80 mV (H) and -120 mV (I). Recordings were carried out in 1 M KCl, 15 mM HEPES, 2 M urea, pH 7.5, 50 kHz sampling and 10 kHz Bessel filter.*

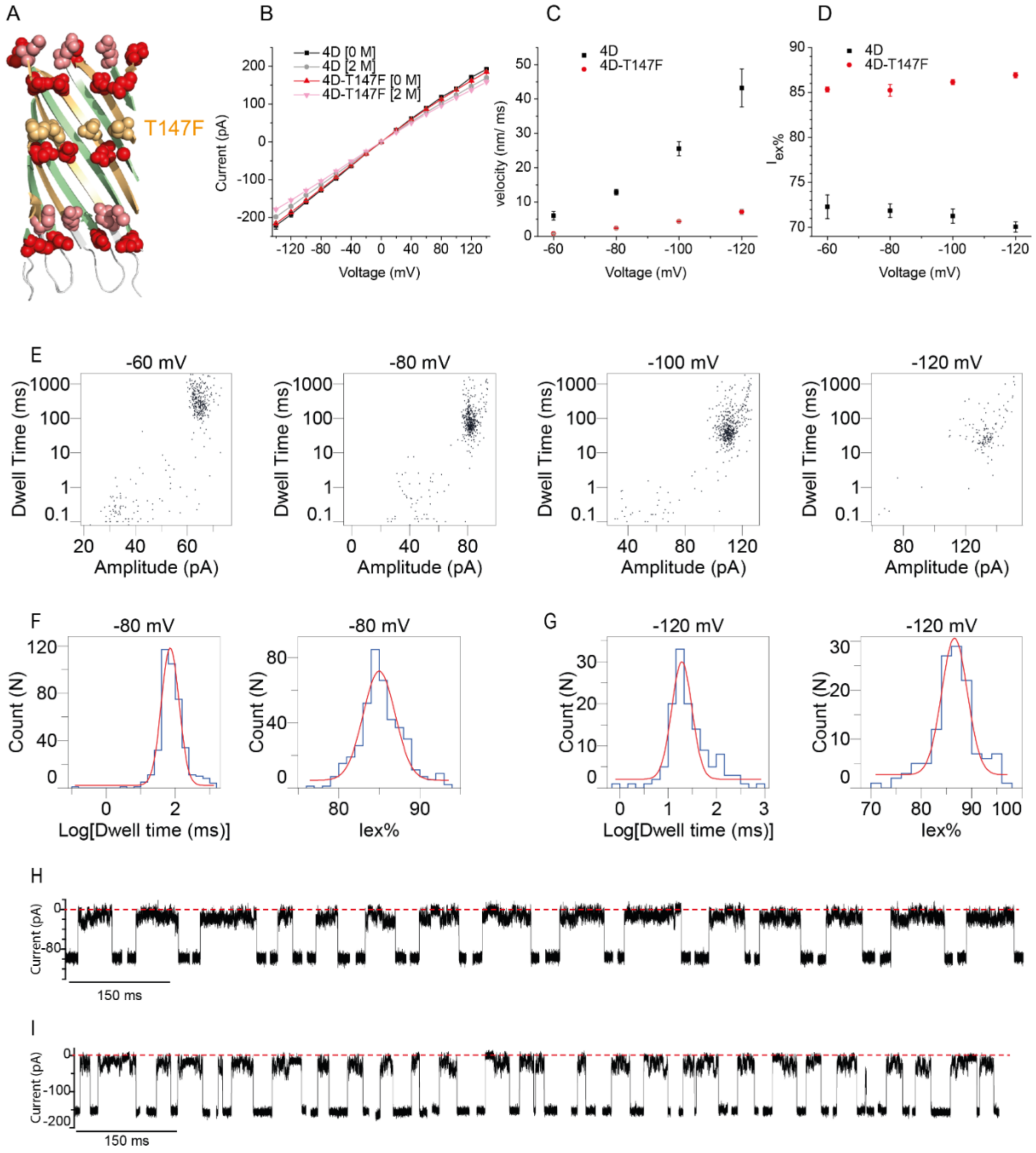


***Figure S11. Translocation of malE219a through the CytK 4D-S149F nanopore. A)*** *The barrel of the CytK nanopore. The N-term strand is depicted in green, while the C-term strand in green. The relevant residues are shown as spheres: E112 and E139 in pink, the four Asp positions in red (K128, Q145, S151 and K155) and S149 in orange.* ***B)*** *IV curves in 1 M KCl at pH 7.5 of the 4D and 4D-S149F mutants in 0 and 2 M urea; each curve was obtained from a triplicate measurement.* ***C)*** *Translocation velocity dependence of malE219a through the 4D and 4D-S149F.* ***D)*** *$I_{ex\%}$ dependence of malE219a translocation through the 4D and 4D-S149F. Data points represent averages from three independent experiments and the error bars correspond to standard deviations (SD).* ***E)*** *Scatter plots (dwell time vs amplitude) associated with the malE219a translocation at the sampled potentials.* ***F-G)*** *Examples of histograms obtained for the log(dwell time) and Iex at -80 mV and -120 mV, respectively.* ***H-I)*** *Typical malE219a translocation events at -80 mV (H) and -120 mV (I). Recordings were carried out in 1 M KCl, 15 mM HEPES, 2 M urea, pH 7.5, 50 kHz sampling and 10 kHz Bessel filter.*

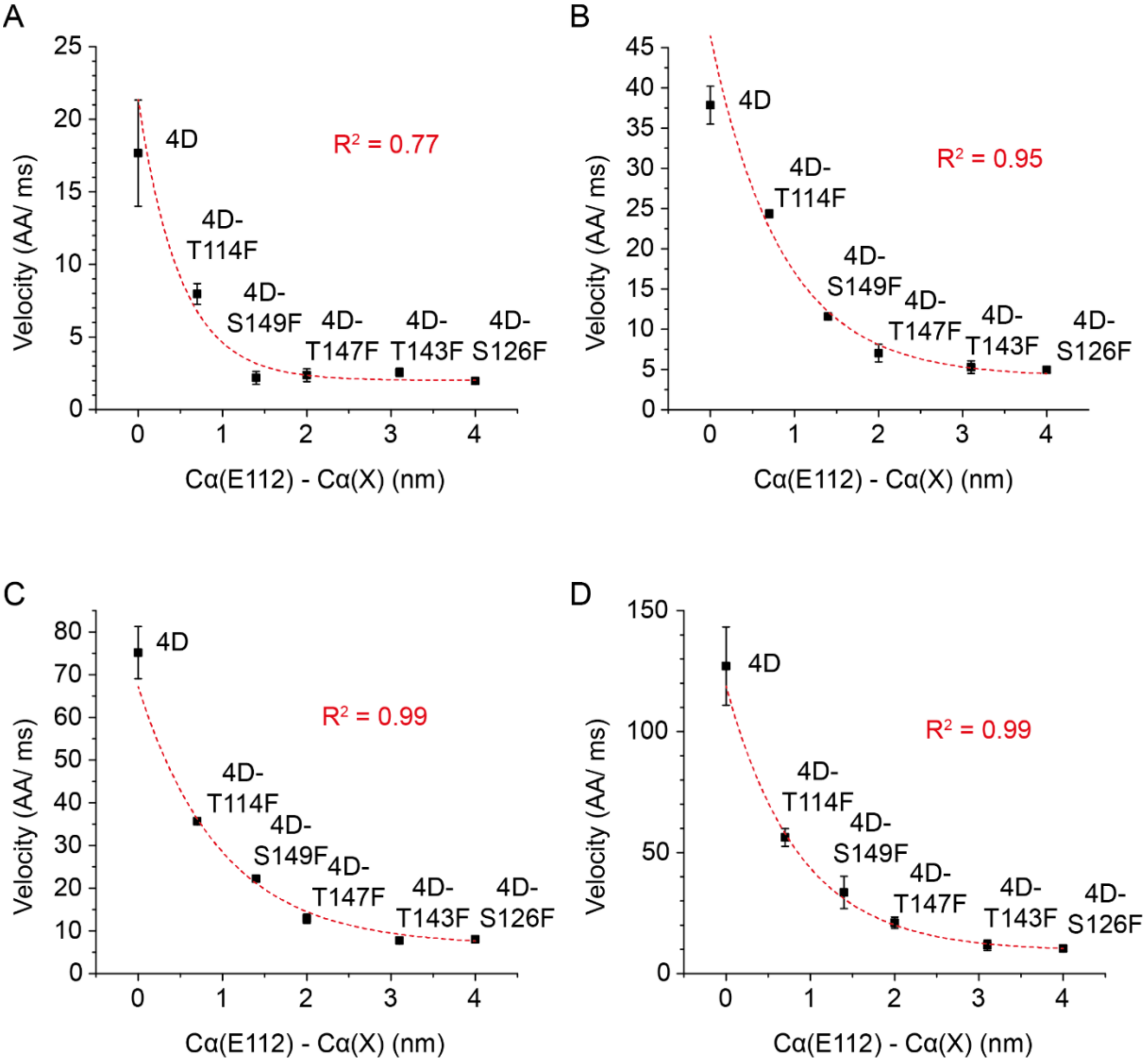


***Figure S12. Exponential fits of the velocities (AAs/ ms) through the CytK-4D-X->F mutants as a function of the distance (Cα-Cα distance between the Phe residue and the entry of the barrel, E112).*** *Different potentials: -60 mV (A), -80 mV (B), -100 mV (C) and -120 mV (D). 0 nm corresponds to the base mutant, CytK 4D where no Phe is introduced.*

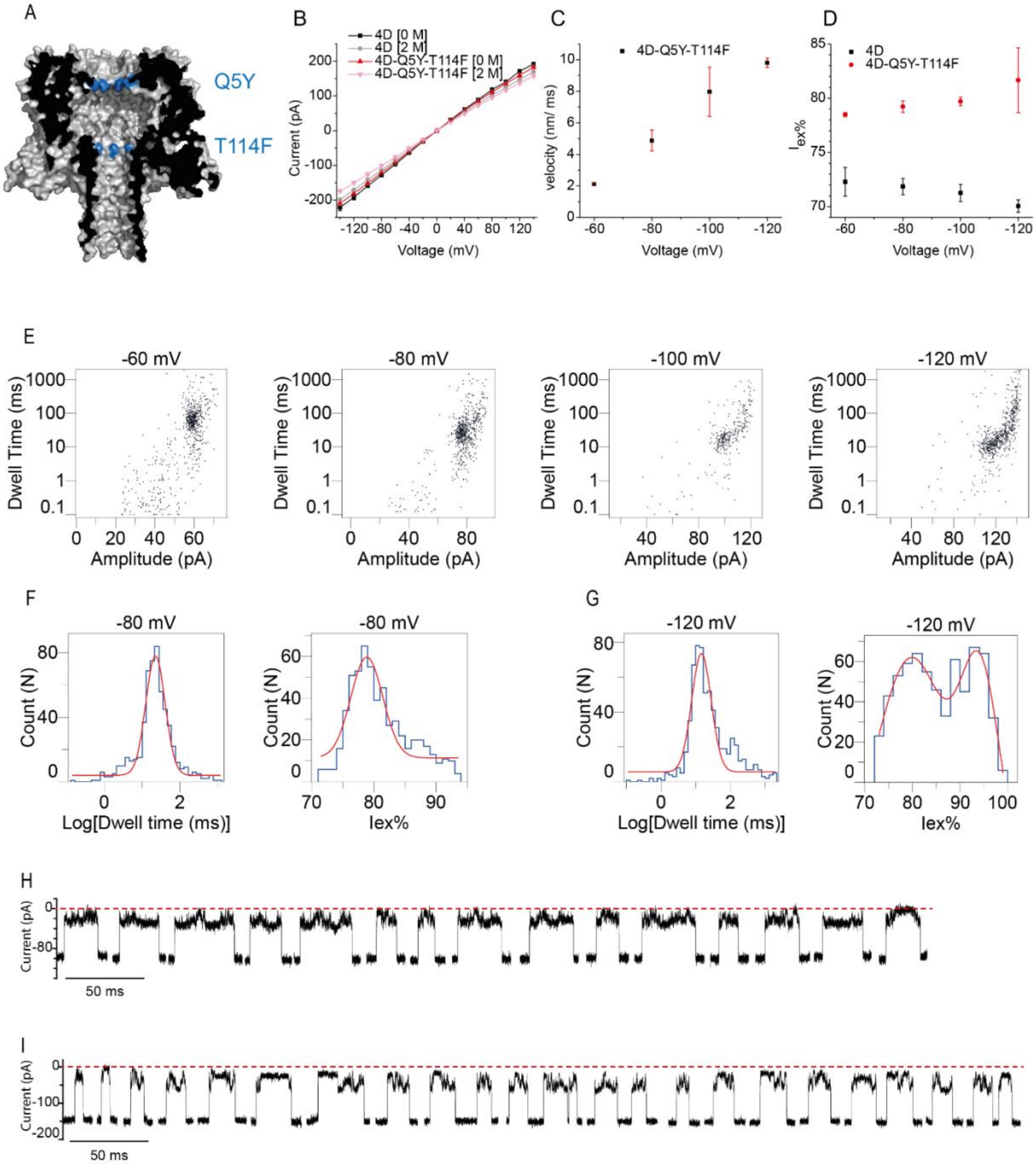


***Figure S13. Translocation of malE219a through the CytK 4D-Q5Y-T114F nanopore.*** ***A)*** *The CytK nanopore, where the Q5 and T114 positions are highlighted.* ***B)*** *IV curves in 1 M KCl at pH 7.5 of the 4D and 4D-Q5Y-T114F mutants in 0 and 2 M urea; each curve was obtained from a triplicate measurement.* ***C)*** *Translocation velocity dependence of malE219a through the 4D and 4D-Q5Y-T114F.* ***D)*** *$I_{ex\%}$ dependence of malE219a translocation through the 4D and 4D-Q5Y-T114F. Data points represent averages from three independent experiments and the error bars correspond to standard deviations (SD).* ***E)*** *Scatter plots (dwell time vs amplitude) associated with the malE219a translocation at the sampled potentials.* ***F-G)*** *Examples of histograms obtained for the log(dwell time) and Iex at -80 mV and -120 mV, respectively.* ***H-I)*** *Typical malE219a translocation events at -80 mV (H) and -120 mV (I). Recordings were carried out in 1 M KCl, 15 mM HEPES, 2 M urea, pH 7.5, 50 kHz sampling and 10 kHz Bessel filter.*

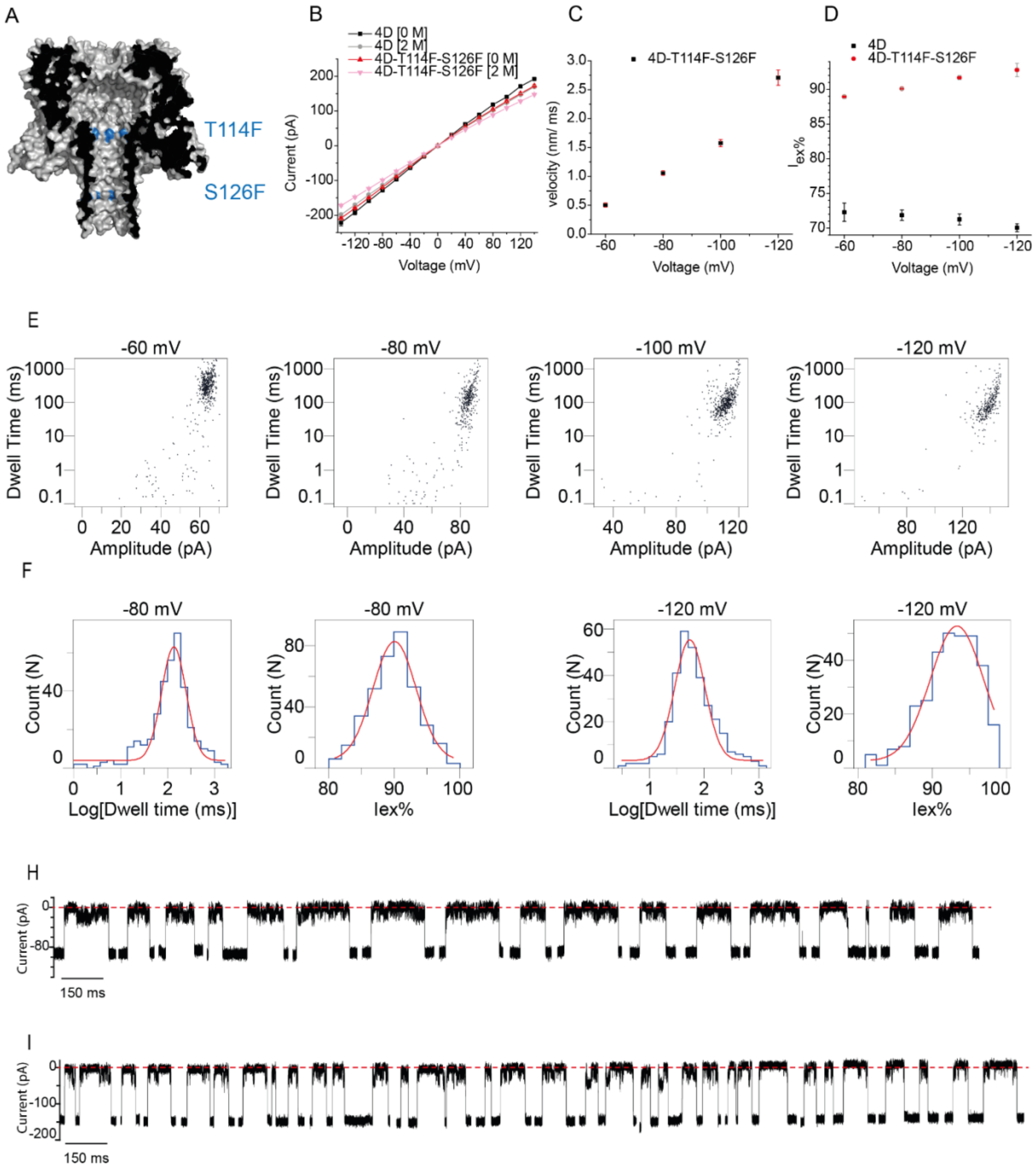


***Figure S14. Translocation of malE219a through the CytK 4D-T114F-S126F nanopore.*** ***A)*** *The CytK nanopore, where the T114 and S126 positions are highlighted.* ***B)*** *IV curves in 1 M KCl at pH 7.5 of the 4D and 4D-T114F-S126F mutants in 0 and 2 M urea; each curve was obtained from a triplicate measurement.* ***C)*** *Translocation velocity dependence of malE219a through the 4D and 4D-T114F-S126F.* ***D)*** *$I_{ex\%}$ dependence of malE219a translocation through the 4D and 4D-T114F-S126F. Data points represent averages from three independent experiments and the error bars correspond to standard deviations (SD).* ***E)*** *Scatter plots (dwell time vs amplitude) associated with the malE219a translocation at the sampled potentials.* ***F-G)*** *Examples of histograms obtained for the log(dwell time) and Iex at -80 mV and -120 mV, respectively.* ***H-I)*** *Typical malE219a translocation events at -80 mV (H) and -120 mV (I). Recordings were carried out in 1 M KCl, 15 mM HEPES, 2 M urea, pH 7.5, 50 kHz sampling and 10 kHz Bessel filter.*

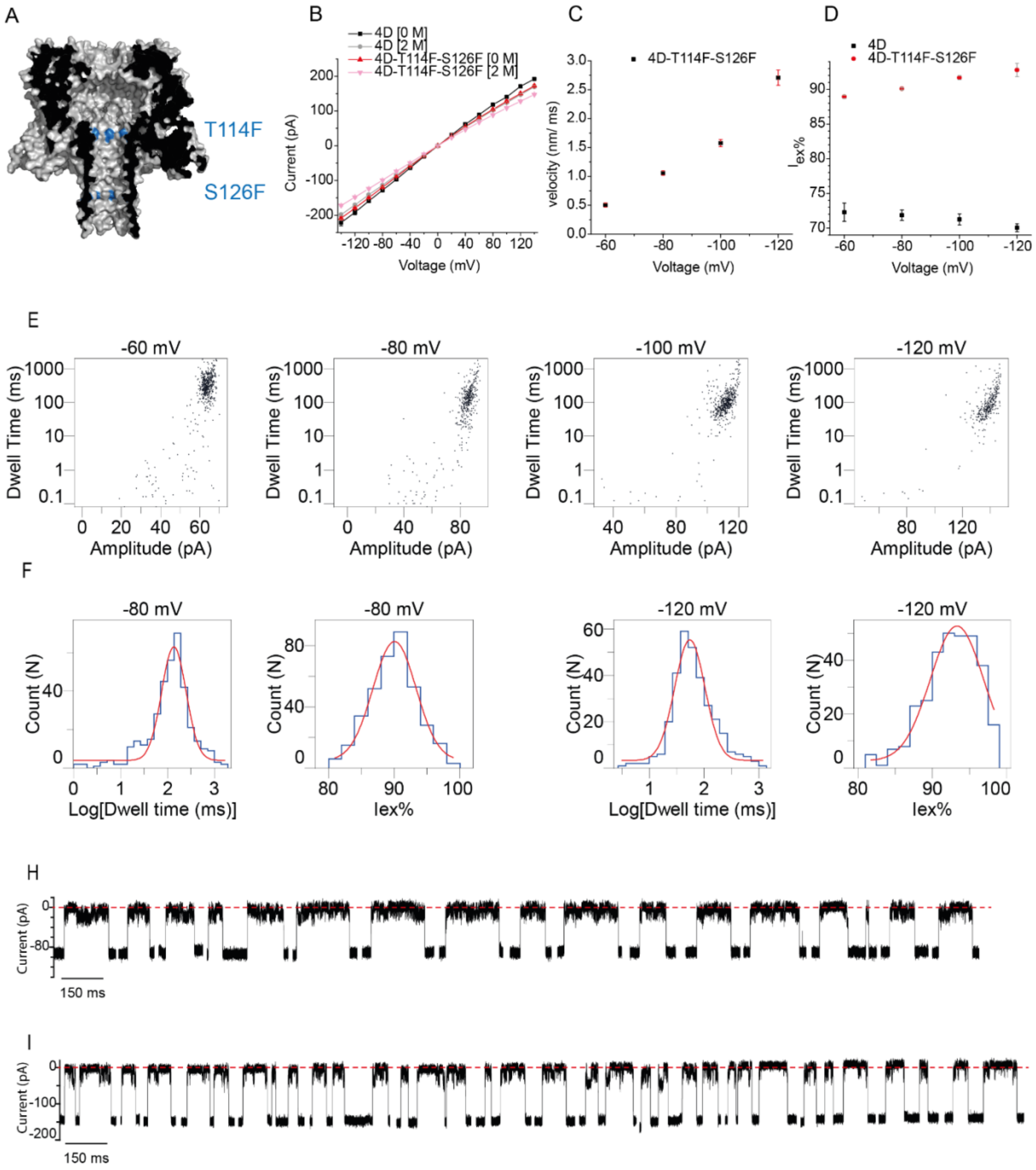

***Figure S15. Translocation of malE219a through the CytK 4D-Q5Y-T114F-S126F nanopore.*** ***A)*** *The CytK nanopore, where the Q5, T114 and S126 positions are highlighted.* ***B)*** *IV curves in 1 M KCl at pH 7.5 of the 4D and 4D-Q5Y-T114F-S126F mutants in 0 and 2 M urea; each curve was obtained from a triplicate measurement.* ***C)*** *Translocation velocity dependence of malE219a through the 4D and 4D-Q5Y-T114F-S126F.* ***D)*** *$I_{ex\%}$ dependence of malE219a translocation through the 4D and 4D-Q5Y-T114F-S126F. Data points represent averages from three independent experiments and the error bars correspond to standard deviations (SD).* ***E)*** *Scatter plots (dwell time vs amplitude) associated with the malE219a translocation at the sampled potentials.* ***F-G)*** *Examples of histograms obtained for the log(dwell time) and Iex at -80 mV and -120 mV, respectively.* ***H-I)*** *Typical malE219a translocation events at -80 mV (H) and -120 mV (I). Recordings were carried out in 1 M KCl, 15 mM HEPES, 2 M urea, pH 7.5, 50 kHz sampling and 10 kHz Bessel filter.*

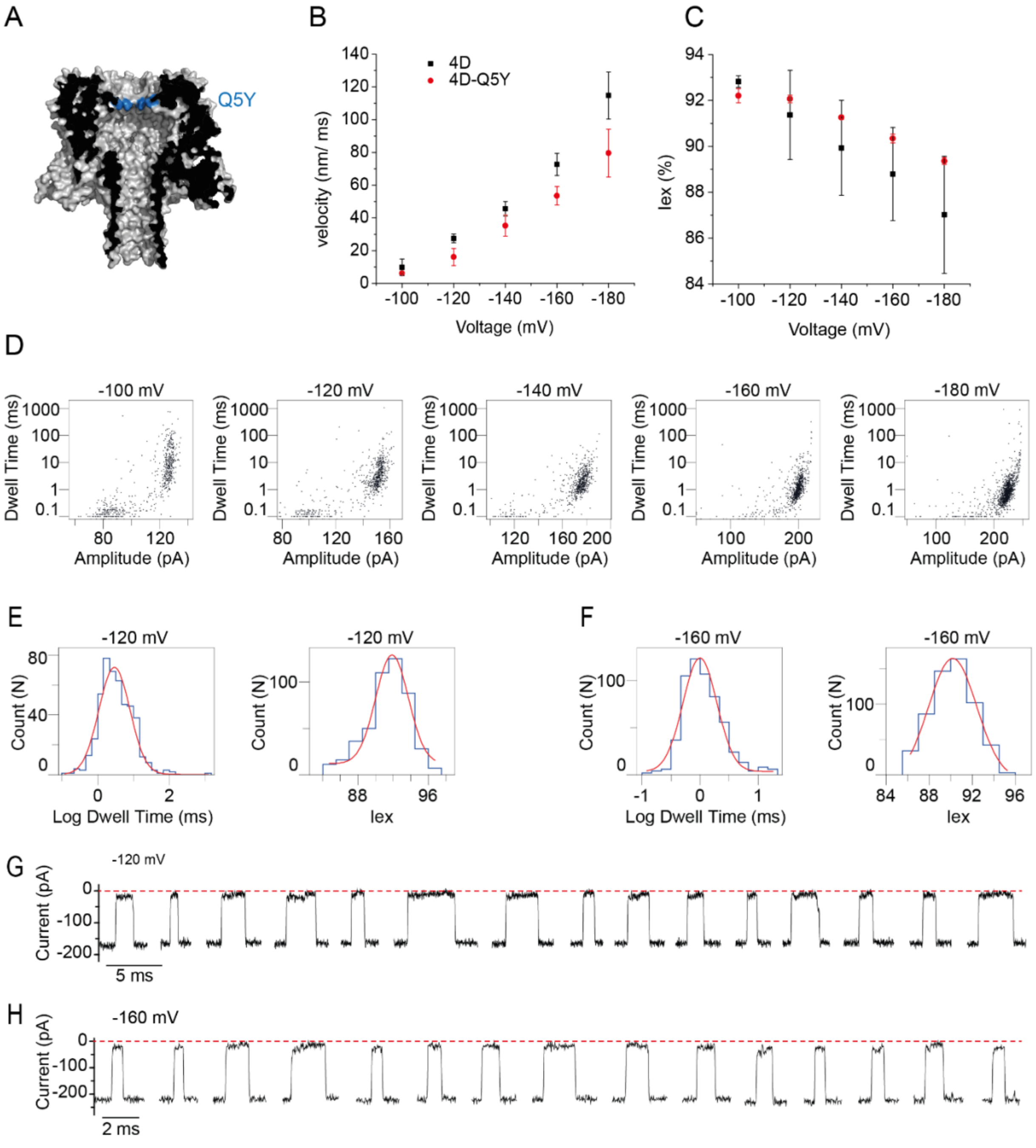


***Figure S16*. *Translocation of tzatziki through the CytK 4D-Q5Y nanopore.*** ***A)*** *The CytK nanopore, where the Q5 position is highlighted.* ***B)*** *Translocation velocity dependence of tzatziki through the 4D and 4D-Q5Y.* ***C)*** *$I_{ex\%}$ dependence of tzatziki translocation through the 4D and 4D-Q5Y. Data points represent averages from three independent experiments and the error bars correspond to standard deviations (SD).* ***D)*** *Scatter plots (dwell time vs amplitude) associated with the tzatziki translocation at the sampled potentials.* ***E-F)*** *Examples of histograms obtained for the log(dwell time) and Iex at -120 mV and -160 mV, respectively.* ***G-H)*** *Typical tzatziki translocation events at -120 mV (G) and -160 mV (H). Recordings were carried out in 1 M KCl, 15 mM HEPES, pH 7.5, 50 kHz sampling and 10 kHz Bessel filter.*

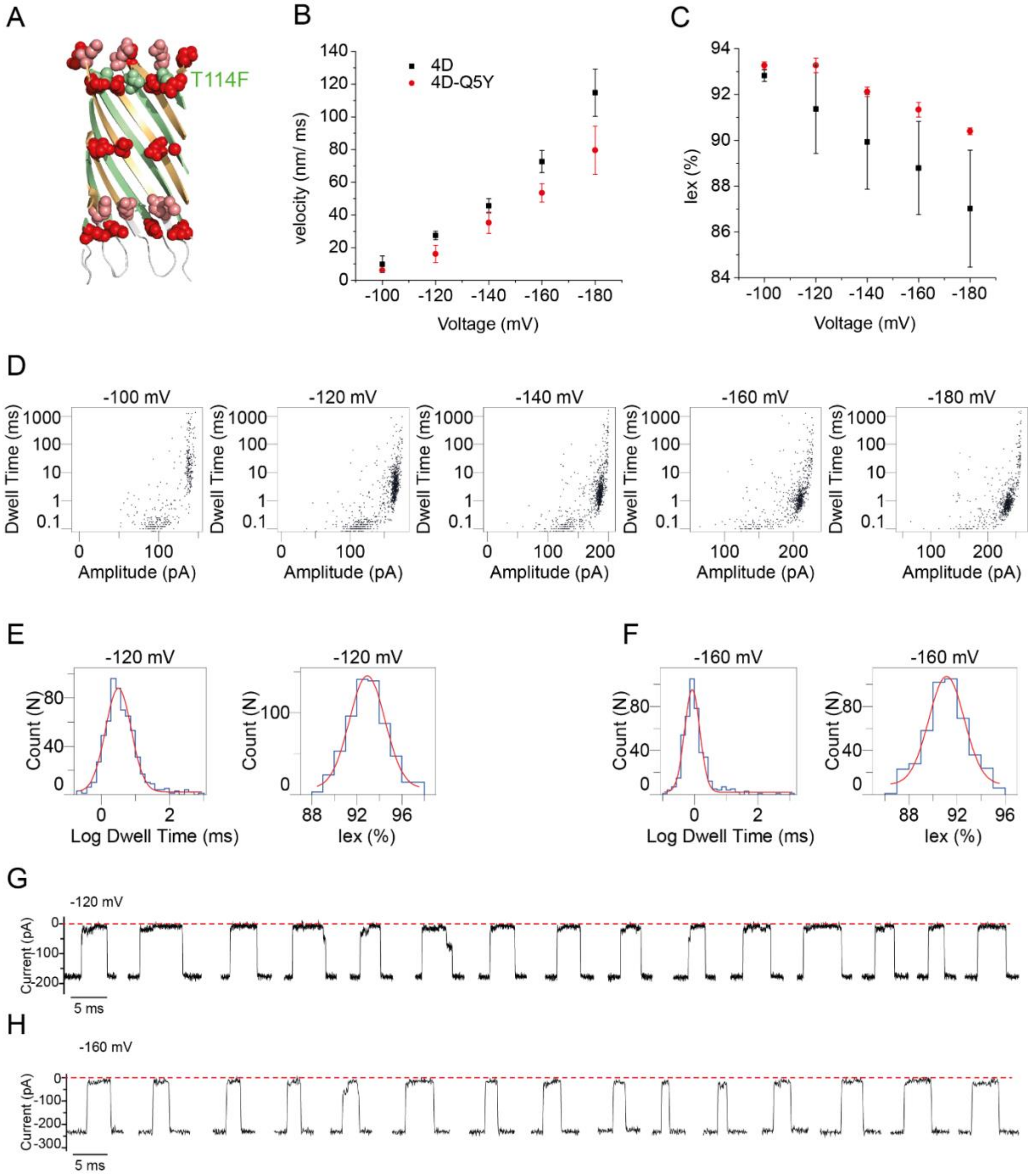


***Figure S17. Translocation of tzatziki through the CytK 4D-T114F nanopore.*** ***A)*** *The barrel of the CytK nanopore. The N-term strand is depicted in green, while the C-term strand in green. The relevant residues are shown as spheres: E112 and E139 in pink, the four Asp positions in red (K128, Q145, S151 and K155) and T114 in green.* ***B)*** *Translocation velocity dependence of tzatziki through the 4D and 4D-T114F.* ***C)*** *$I_{ex\%}$ dependence of tzatziki translocation through the 4D and 4D-T114F. Data points represent averages from three independent experiments and the error bars correspond to standard deviations (SD).* ***D)*** *Scatter plots (dwell time vs amplitude) associated with the tzatziki translocation at the sampled potentials.* ***E-F)*** *Examples of histograms obtained for the log(dwell time) and Iex at -120 mV and -160 mV, respectively.* ***G-H)*** *Typical tzatziki translocation events at -120 mV (G) and -160 mV (H). Recordings were carried out in 1 M KCl, 15 mM HEPES, pH 7.5, 50 kHz sampling and 10 kHz Bessel filter.*

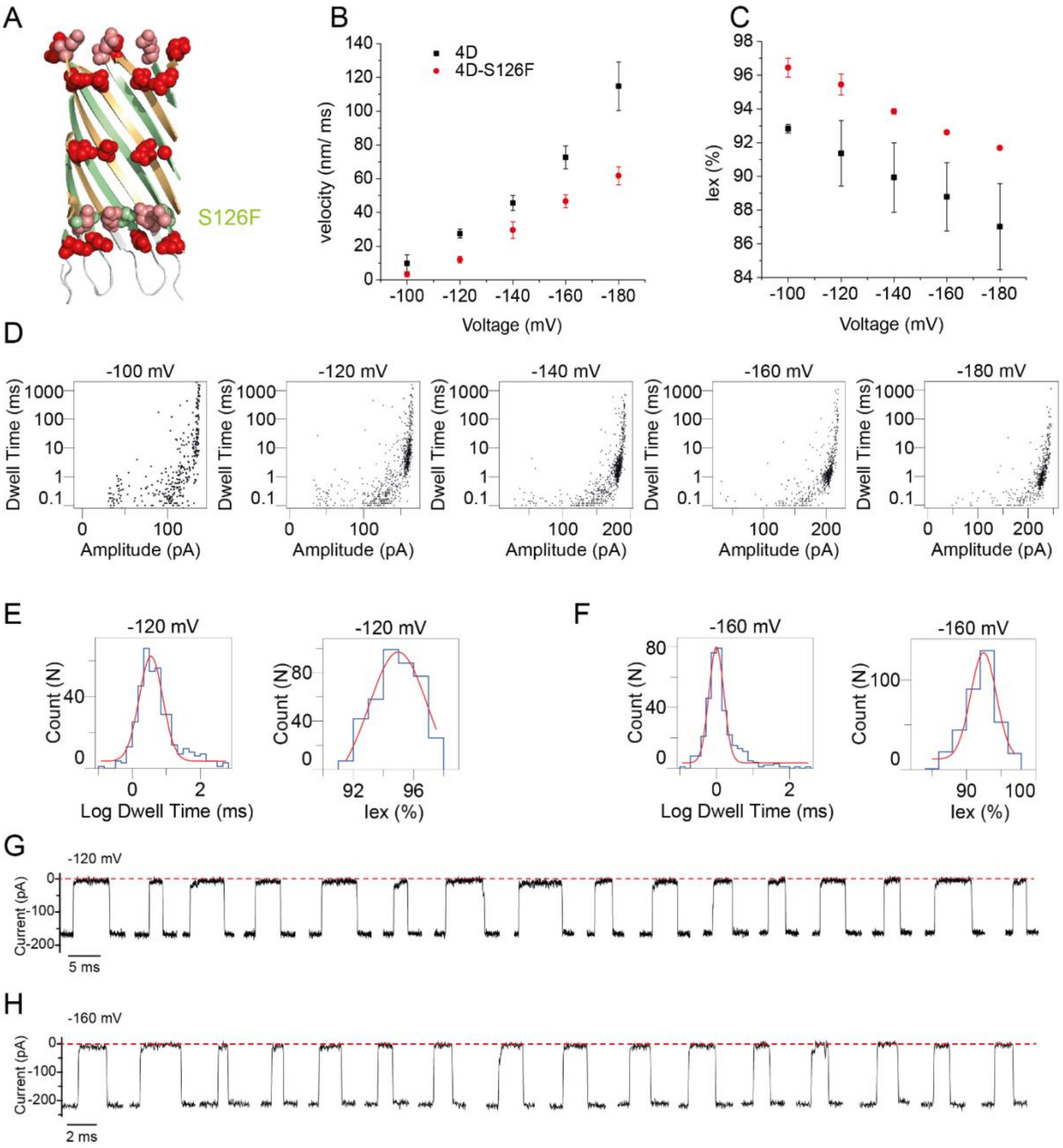


***Figure S18. Translocation of tzatziki through the CytK 4D-S126F nanopore.*** ***A)*** *The barrel of the CytK nanopore. The N-term strand is depicted in green, while the C-term strand in green. The relevant residues are shown as spheres: E112 and E139 in pink, the four Asp positions in red (K128, Q145, S151 and K155) and S126 in green.* ***B)*** *Translocation velocity dependence of tzatziki through the 4D and 4D-S126F.* ***C)*** *$I_{ex\%}$ dependence of tzatziki translocation through the 4D and 4D-S126F. Data points represent averages from three independent experiments and the error bars correspond to standard deviations (SD).* ***D)*** *Scatter plots (dwell time vs amplitude) associated with the tzatziki translocation at the sampled potentials.* ***E-F)*** *Examples of histograms obtained for the log(dwell time) and Iex at -120 mV and -160 mV, respectively.* ***G-H)*** *Typical tzatziki translocation events at -120 mV (G) and -160 mV (H). Recordings were carried out in 1 M KCl, 15 mM HEPES, pH 7.5, 50 kHz sampling and 10 kHz Bessel filter.*

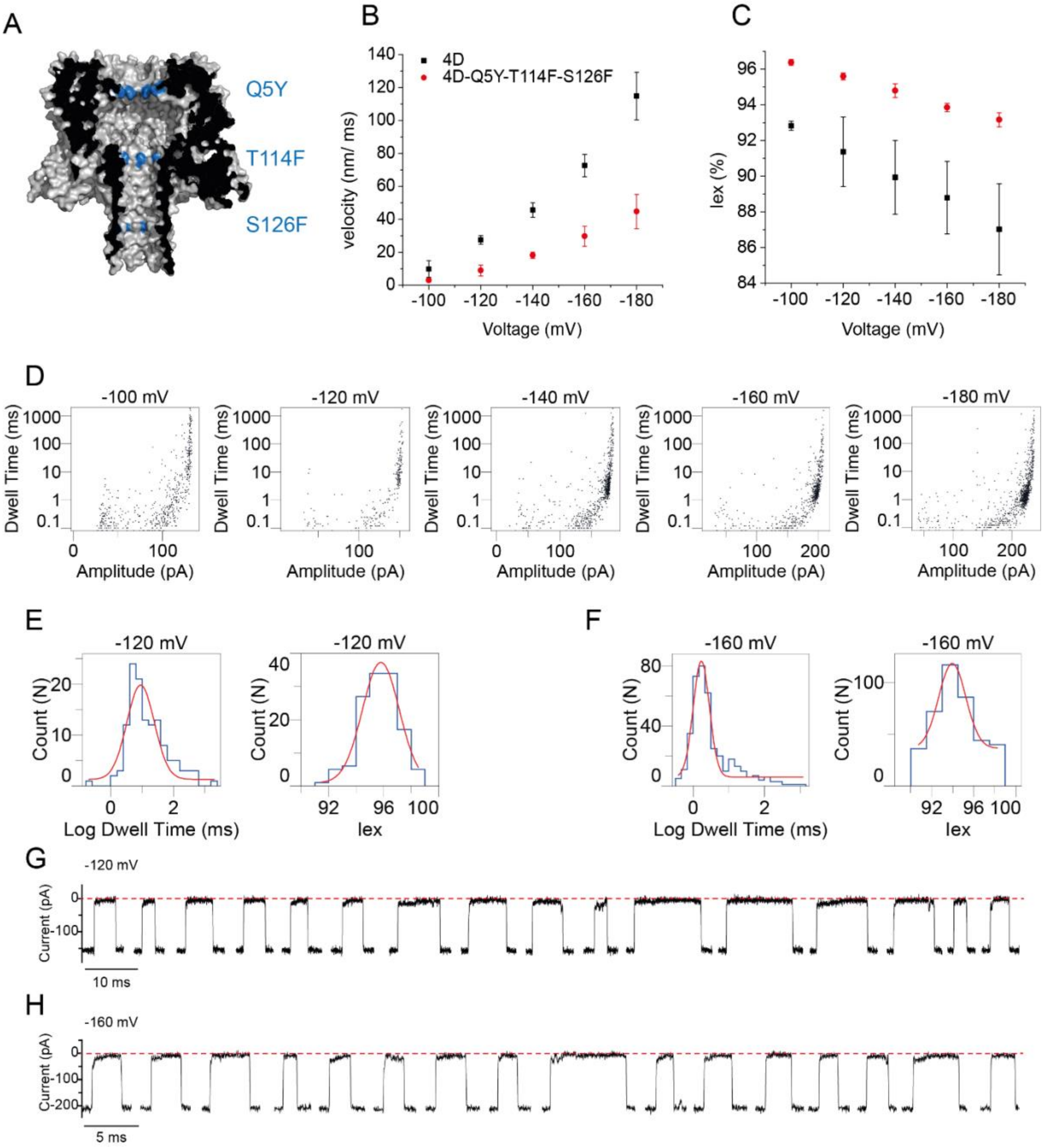


***Figure S19. Translocation of tzatziki through the CytK 4D-Q5Y-T114F-S126F nanopore. A)*** *The CytK nanopore, where the Q5, T114F and S126F positions are highlighted.* ***B)*** *Translocation velocity dependence of tzatziki through the 4D and 4D-Q5Y-T114F-S126F.* ***C)*** *$I_{ex\%}$ dependence of tzatziki translocation through the 4D and 4D-Q5Y-T114F-S126F. Data points represent averages from three independent experiments and the error bars correspond to standard deviations (SD).* ***D)*** *Scatter plots (dwell time vs amplitude) associated with the tzatziki translocation at the sampled potentials.* ***E-F)*** *Examples of histograms obtained for the log(dwell time) and Iex at -120 mV and -160 mV, respectively.* ***G-H)*** *Typical tzatziki translocation events at -120 mV (G) and -160 mV (H). Recordings were carried out in 1 M KCl, 15 mM HEPES, pH 7.5, 50 kHz sampling and 10 kHz Bessel filter.*

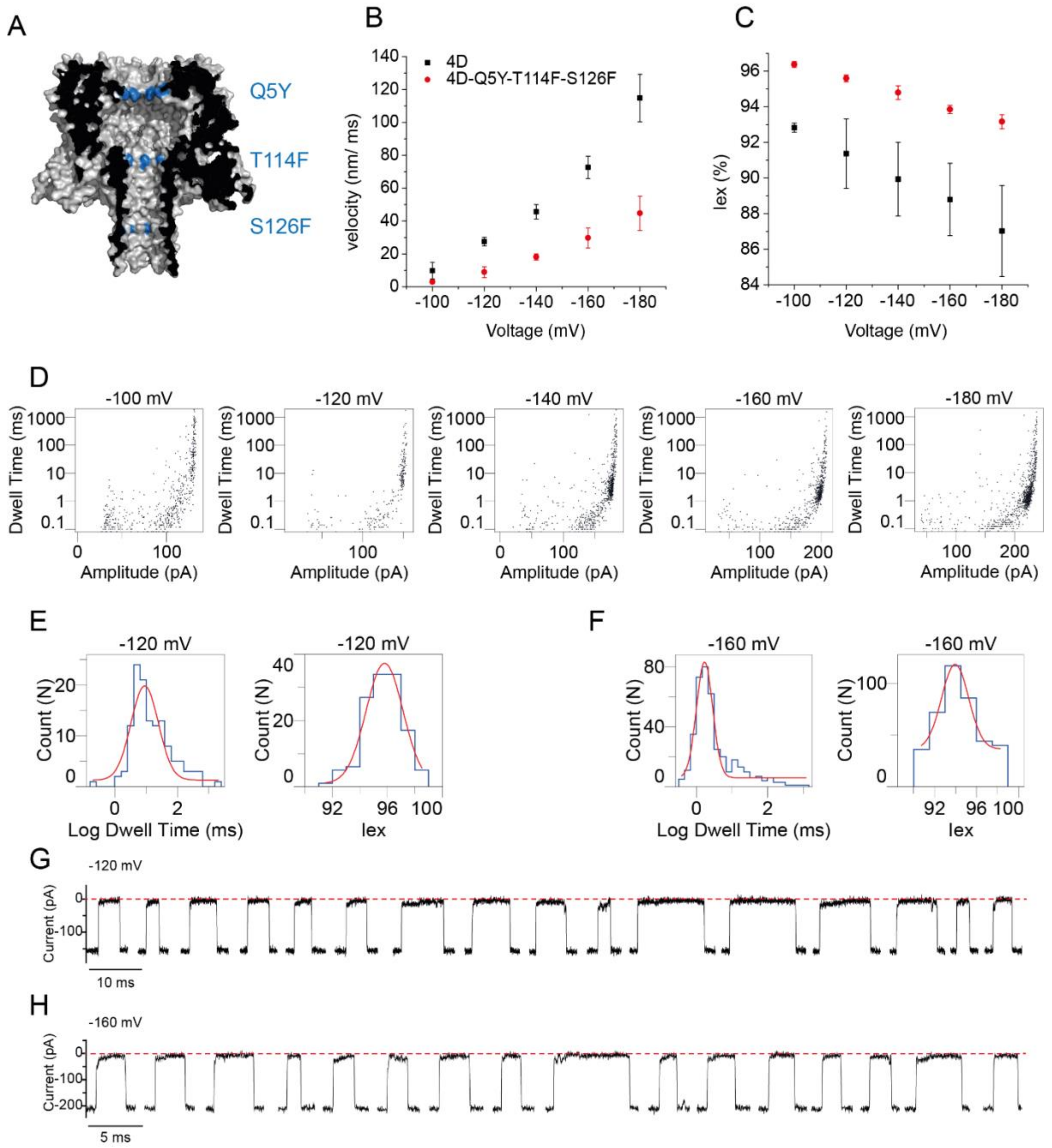


***Figure S20. Translocation of malE219a through the CytK 4D-S126I nanopore. A)*** *The barrel of the CytK nanopore. The N-term strand is depicted in green, while the C-term strand in green. The relevant residues are shown as spheres: E112 and E139 in pink, the four Asp positions in red (K128, Q145, S151 and K155) and S126 in green.* ***B)*** *IV curves in 1 M KCl at pH 7.5 of the 4D and 4D-S126I mutants in 0 and 2 M urea; each curve was obtained from a triplicate measurement.* ***C)*** *Translocation velocity dependence of malE219a through the 4D and 4D-S126I.* ***D)*** *$I_{ex\%}$ dependence of malE219a translocation through the 4D and 4D-S126F. Data points represent averages from three independent experiments and the error bars correspond to standard deviations (SD).* ***E)*** *Scatter plots (dwell time vs amplitude) associated with the malE219a translocation at the sampled potentials.* ***F-G)*** *Examples of histograms obtained for the log(dwell time) and Iex at -80 mV and -120 mV, respectively.* ***H-I)*** *Typical malE219a translocation events at -80 mV (H) and -120 mV (I). Recordings were carried out in 1 M KCl, 15 mM HEPES, 2 M urea, pH 7.5, 50 kHz sampling and 10 kHz Bessel filter.*

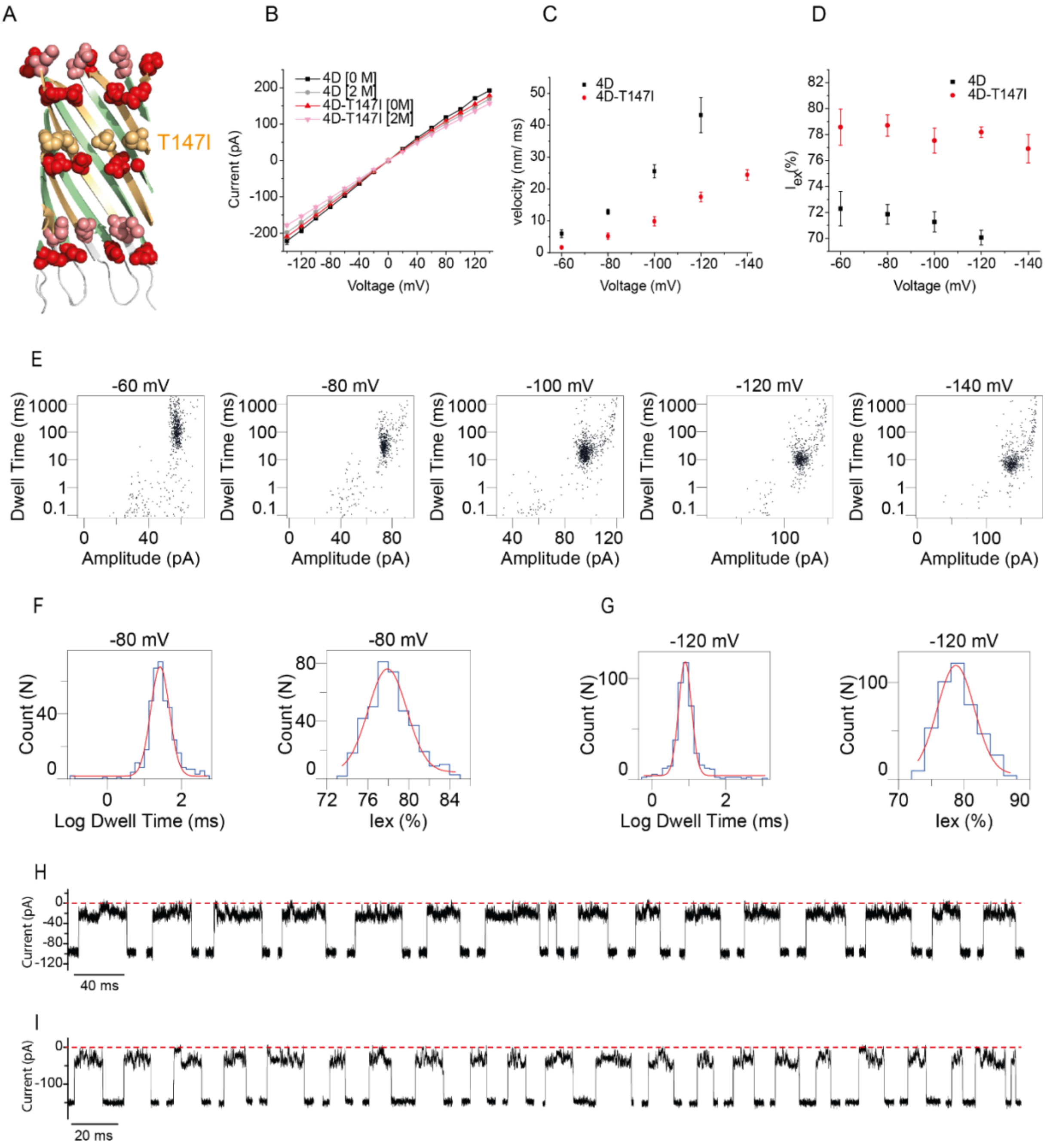


***Figure S21. Translocation of malE219a through the CytK 4D-T147I nanopore. A)*** *The barrel of the CytK nanopore. The N-term strand is depicted in green, while the C-term strand in green. The relevant residues are shown as spheres: E112 and E139 in pink, the four Asp positions in red (K128, Q145, S151 and K155) and T147 in orange.* ***B)*** *IV curves in 1 M KCl at pH 7.5 of the 4D and 4D-T147I mutants in 0 and 2 M urea; each curve was obtained from a triplicate measurement.* ***C)*** *Translocation velocity dependence of malE219a through the 4D and 4D-T147I.* ***D)*** *$I_{ex\%}$ dependence of malE219a translocation through the 4D and 4D-T147F. Data points represent averages from three independent experiments and the error bars correspond to standard deviations (SD).* ***E)*** *Scatter plots (dwell time vs amplitude) associated with the malE219a translocation at the sampled potentials.* ***F-G)*** *Examples of histograms obtained for the log(dwell time) and Iex at -80 mV and -120 mV, respectively.* ***H-I)*** *Typical malE219a translocation events at -80 mV (H) and -120 mV (I). Recordings were carried out in 1 M KCl, 15 mM HEPES, 2 M urea, pH 7.5, 50 kHz sampling and 10 kHz Bessel filter.*

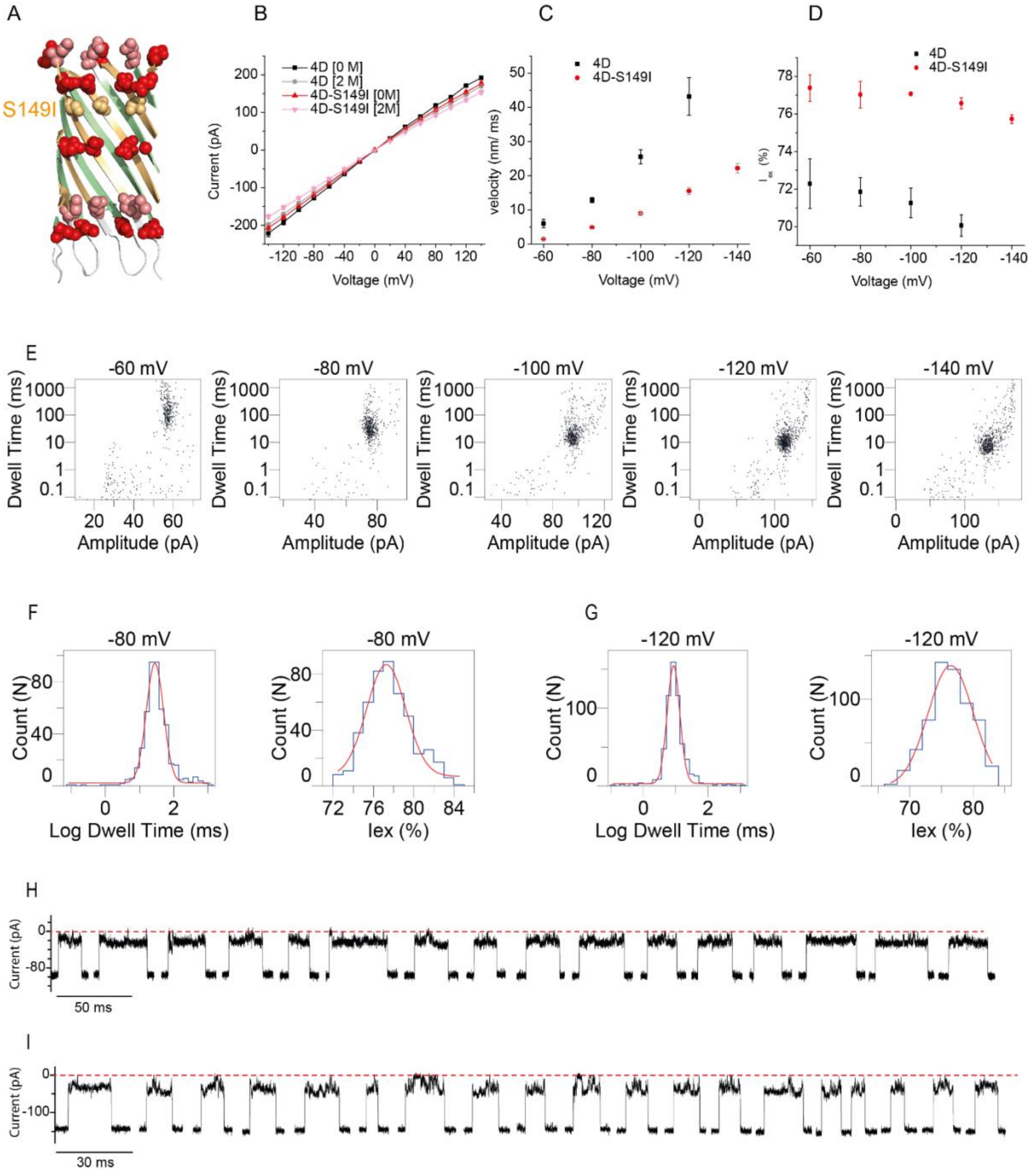


***Figure S22. Translocation of malE219a through the CytK 4D-S149I nanopore.*** ***A)*** *The barrel of the CytK nanopore. The N-term strand is depicted in green, while the C-term strand in green. The relevant residues are shown as spheres: E112 and E139 in pink, the four Asp positions in red (K128, Q145, S151 and K155) and S149 in orange.* ***B)*** *IV curves in 1 M KCl at pH 7.5 of the 4D and 4D-S149I mutants in 0 and 2 M urea; each curve was obtained from a triplicate measurement.* ***C)*** *Translocation velocity dependence of malE219a through the 4D and 4D-S149I.* ***D)*** *$I_{ex\%}$ dependence of malE219a translocation through the 4D and 4D-S149F. Data points represent averages from three independent experiments and the error bars correspond to standard deviations (SD).* ***E)*** *Scatter plots (dwell time vs amplitude) associated with the malE219a translocation at the sampled potentials.* ***F-G)*** *Examples of histograms obtained for the log(dwell time) and Iex at -80 mV and -120 mV, respectively.* ***H-I)*** *Typical malE219a translocation events at -80 mV (H) and -120 mV (I). Recordings were carried out in 1 M KCl, 15 mM HEPES, 2 M urea, pH 7.5, 50 kHz sampling and 10 kHz Bessel filter.*

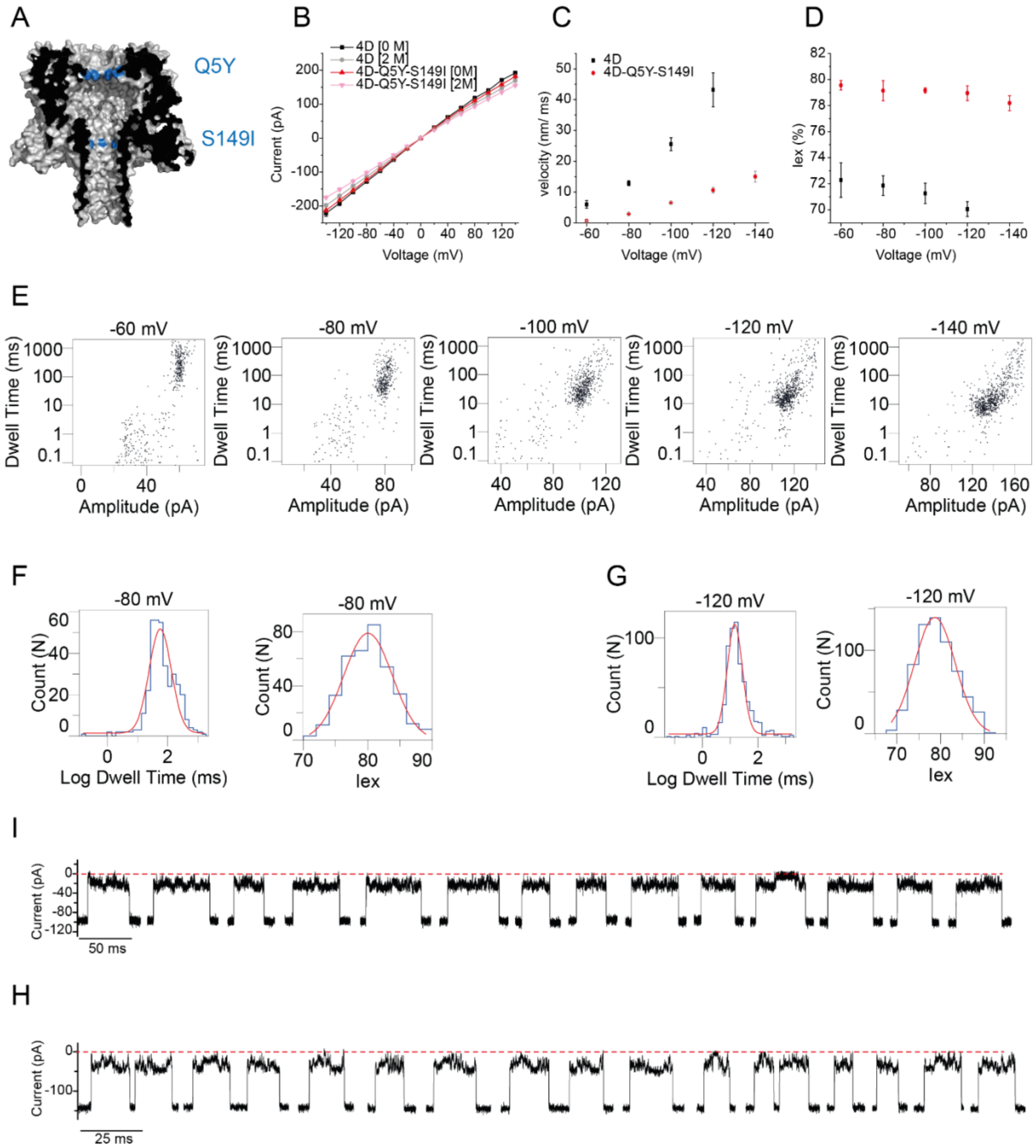


***Figure S23. Translocation of malE219a through the CytK 4D-Q5Y-S149I nanopore. A)*** *The CytK nanopore, where the Q5 and S149 positions are highlighted.* ***B)*** *IV curves in 1 M KCl at pH 7.5 of the 4D and 4D-Q5Y-S149I mutants in 0 and 2 M urea; each curve was obtained from a triplicate measurement.* ***C)*** *Translocation velocity dependence of malE219a through the 4D and 4D-Q5Y-S149I.* ***D)*** *$I_{ex\%}$ dependence of malE219a translocation through the 4D and 4D-Q5Y-S149I. Data points represent averages from three independent experiments and the error bars correspond to standard deviations (SD).* ***E)*** *Scatter plots (dwell time vs amplitude) associated with the malE219a translocation at the sampled potentials.* ***F-G)*** *Examples of histograms obtained for the log(dwell time) and Iex at -80 mV and -120 mV, respectively.* ***H-I)*** *Typical malE219a translocation events at -80 mV (H) and -120 mV (I). Recordings were carried out in 1 M KCl, 15 mM HEPES, 2 M urea, pH 7.5, 50 kHz sampling and 10 kHz Bessel filter.*

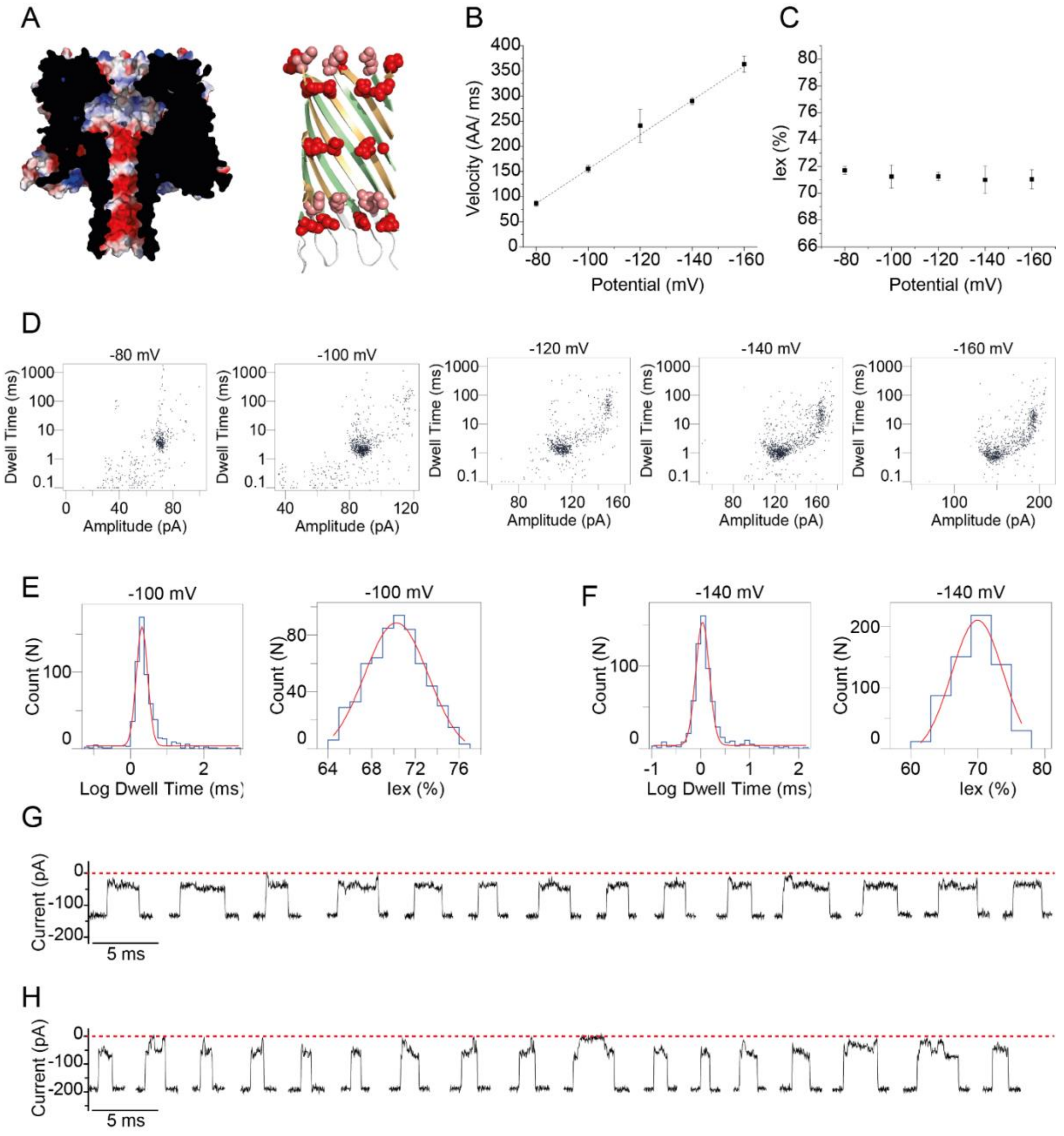


I

MADTRIGVTIYKYDDNFMSVVRKAIEQDAKAAPDVQLLMNDSQNDQSKQNDQIDVLLAKG
VKALAINLVDPAAAGTVIEKARGQNVPVVFFNKEPSRKALDSYDKAYYVGTDSKESGIIQ
GDLIAKHWAANQGWDLNKDGQIQFVLLKGEPGAPDAEARTTYVIKELNDKGIKTEQLQLD
TAMWDTAQAKDKMDAWLSGPNANKIEVVIANNDAMAMGAVEALKAHNKSSIPVFGVDALP
EALALVKSGALAGTVLNDANNQAKATFDLAKNLADGKGAADGTNWKIDNKVVRVPYVGVD
KDNLAEFSKKGSSHHHHHH

***Figure S24. Translocation of GBP-H152A Δperi through the CytK 4D nanopore.*** ***A)*** *The CytK-4D nanopore, shown as electrostatic map and a barrel close-up.* ***B)*** *Translocation velocity dependence of GBP-H152A Δperi through the CytK-4D.* ***C)*** *$I_{ex\%}$ dependence of GBP-H152A Δperi translocation through the CytK-4D. Data points represent averages from three independent experiments and the error bars correspond to standard deviations (SD).* ***D)*** *Scatter plots (dwell time vs amplitude) associated with the GBP-H152A Δperi translocation at the sampled potentials.* ***E-F)*** *Examples of histograms obtained for the log(dwell time) and Iex at -100 mV and -140 mV, respectively.* ***G-H)*** *Typical GBP-H152A Δperi translocation events at -100 mV (G) and -140 mV (H). Recordings were carried out in 1 M KCl, 15 mM HEPES, 2.6 M urea, pH 7.5, 50 kHz sampling and 10 kHz Bessel filter.* ***I)*** *Protein sequence of GBP-H152A Δperi.*

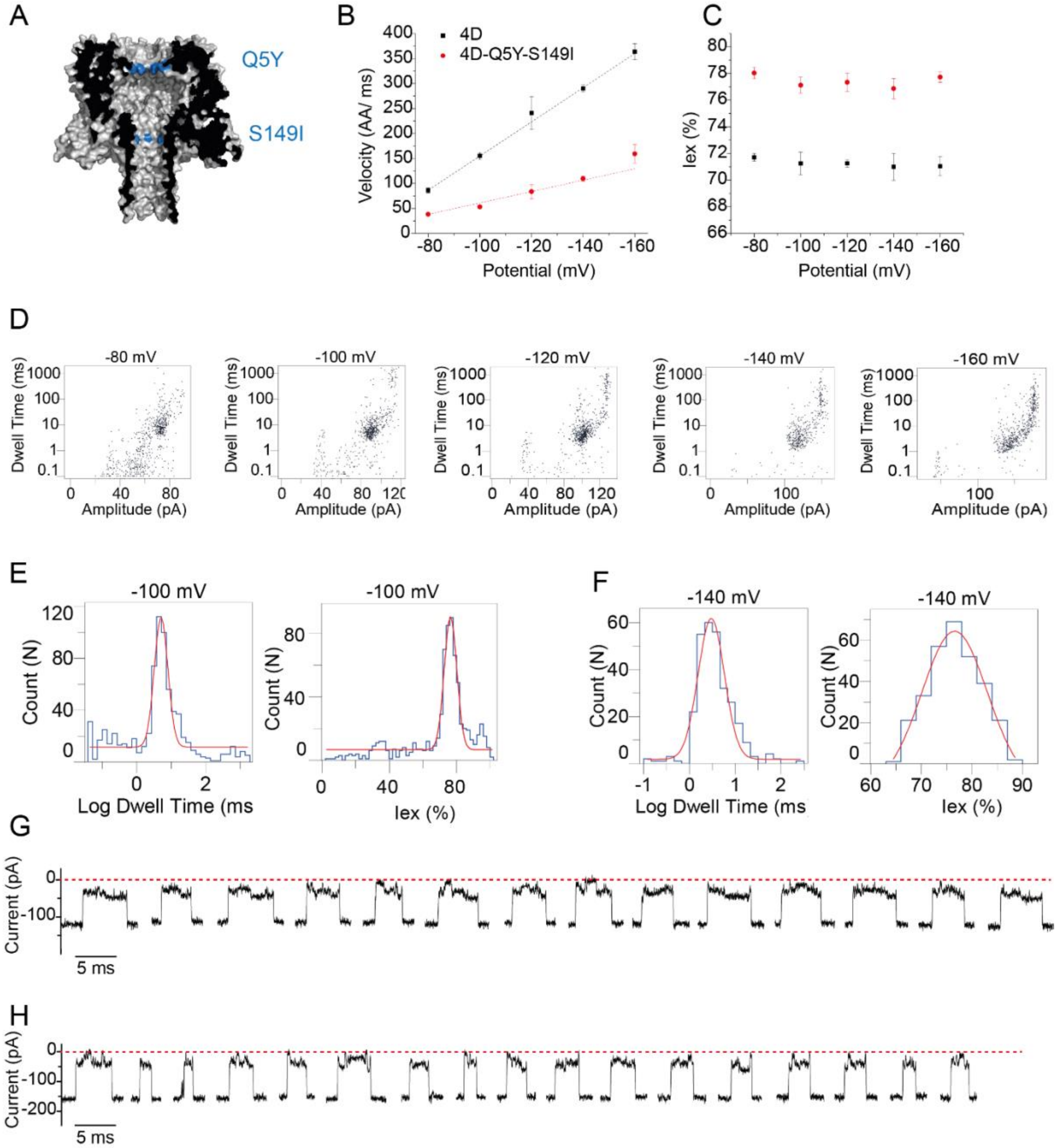


I

MADTRIGVTIYKYDDNFMSVVRKAIEQDAKAAPDVQLLMNDSQNDQSKQNDQIDVLLAKG
VKALAINLVDPAAAGTVIEKARGQNVPVVFFNKEPSRKALDSYDKAYYVGTDSKESGIIQ
GDLIAKHWAANQGWDLNKDGQIQFVLLKGEPGAPDAEARTTYVIKELNDKGIKTEQLQLD
TAMWDTAQAKDKMDAWLSGPNANKIEVVIANNDAMAMGAVEALKAHNKSSIPVFGVDALP
EALALVKSGALAGTVLNDANNQAKATFDLAKNLADGKGAADGTNWKIDNKVVRVPYVGVD
KDNLAEFSKKGSSHHHHHH

***Figure S25. Translocation of GBP-H152A Δperi through the CytK 4D-Q5Y-S149I nanopore. A)*** *The CytK nanopore, where the Q5 and S149 positions are highlighted.* ***B)*** *Translocation velocity dependence of GBP-H152A Δperi through the CytK-4D and CytK 4D-Q5Y-S149I.* ***C)*** *$I_{ex\%}$ dependence of GBP-H152A Δperi translocation through the CytK-4D and CytK 4D-Q5Y-S149I. Data points represent averages from three independent experiments and the error bars correspond to standard deviations (SD).* ***D)*** *Scatter plots (dwell time vs amplitude) associated with the GBP-H152A Δperi translocation at the sampled potentials.* ***E-F)*** *Examples of histograms obtained for the log(dwell time) and Iex at -100 mV and -140 mV, respectively.* ***G-H)*** *Typical GBP-H152A Δperi translocation events at -100 mV (G) and -140 mV (H). Recordings were carried out in 1 M KCl, 15 mM HEPES, 2.6 M urea, pH 7.5, 50 kHz sampling and 10 kHz Bessel filter.* ***I)*** *Protein sequence of GBP-H152A Δperi.*

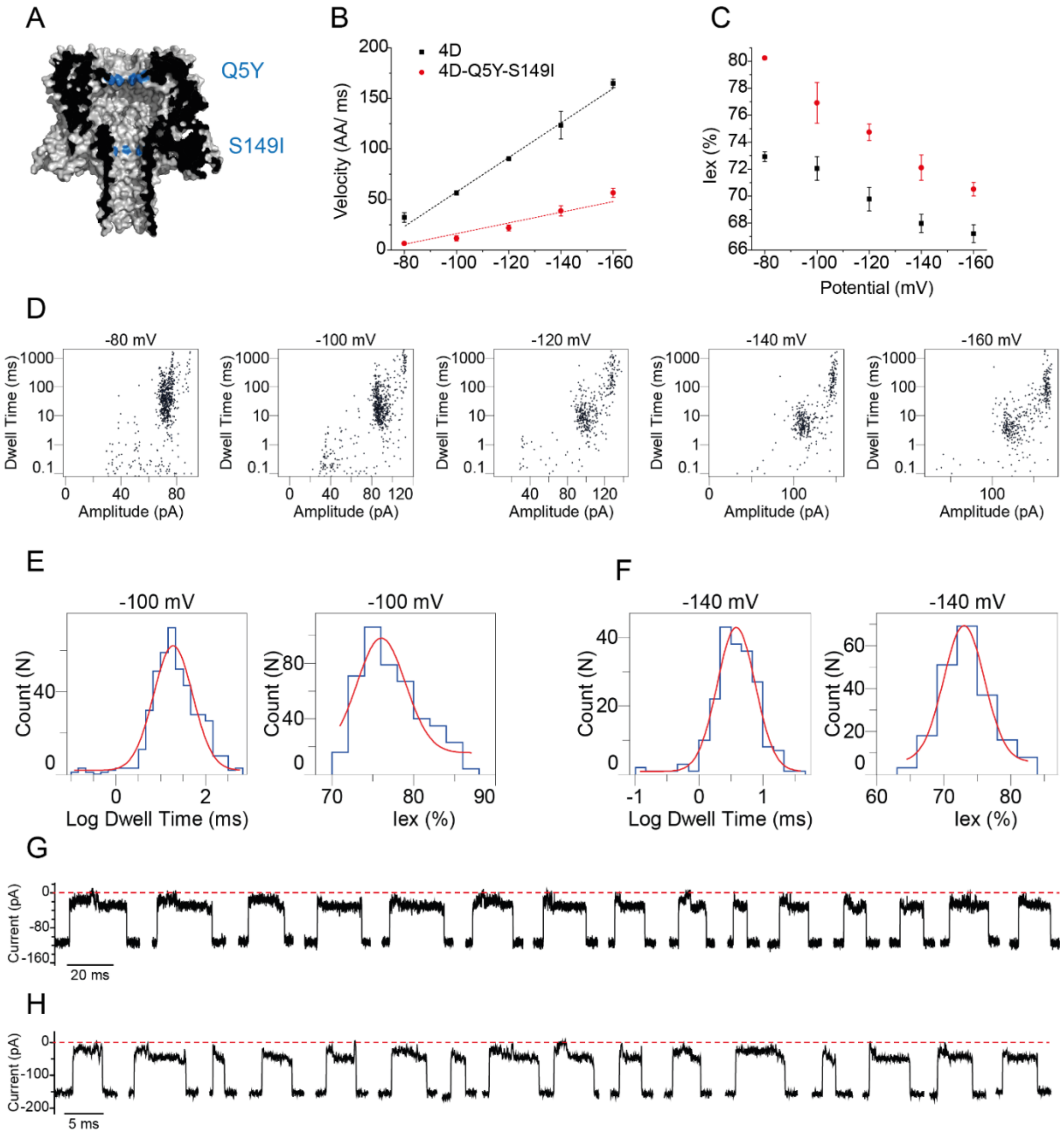


I

MAHHHHHHGSAMISLIAALAVDRVIGMENAMPWNLPADLAGFKRNTLDKPVIMGRHTWESIGRPLPGRKNIILSSQPGTDDRVT
WVKSVDEAIAAAGDVPEIMVIGGGRVYEQFLPKAQKLYLTHIDAEVEGDTHFPDYEPDDLESVFSEFHDADAQNSHSYSFEILERR

***Figure S26. Translocation of DHFR-W30G-W133L through the CytK 4D-Q5Y-S149I nanopore.*** ***A)*** *The CytK nanopore, where the Q5 and S149 positions are highlighted.* ***B)*** *Translocation velocity dependence of DHFR-W30G-W133L through the 4D and 4D-Q5Y-S149I.* ***C)*** *$I_{ex\%}$ dependence of DHFR-W30G-W133L translocation through the 4D and 4D-Q5Y-S149I. Data points represent averages from three independent experiments and the error bars correspond to standard deviations (SD).* ***D)*** *Scatter plots (dwell time vs amplitude) associated with the DHFR-W30G-W133L translocation at the sampled potentials.* ***E-F)*** *Examples of histograms obtained for the log(dwell time) and Iex at -80 mV and -120 mV, respectively.* ***G-H)*** *Typical malE219a translocation events at -80 mV (G) and -120 mV (H). Recordings were carried out in 1 M KCl, 15 mM HEPES, 2.6 M urea, pH 7.5, 50 kHz sampling and 10 kHz Bessel filter.* ***I)*** *Protein sequence of DHFR-W30G-W133L.*

**References**


1. Bitinaite, J. *et al.* USERTM friendly DNA engineering and cloning method by uracil excision. *Nucleic Acids Res* **35**, 1992–2002 (2007).

2. Nørholm, M. H. A mutant Pfu DNA polymerase designed for advanced uracil-excision DNA engineering. *BMC Biotechnol* **10**, 21 (2010).

3. Cavaleiro, A. M., Kim, S. H., Seppälä, S., Nielsen, M. T. & Nørholm, M. H. H. Accurate DNA Assembly and Genome Engineering with Optimized Uracil Excision Cloning. *ACS Synth Biol* **4**, 1042–1046 (2015).

4. Sauciuc, A., Morozzo della Rocca, B., Tadema, M. J., Chinappi, M. & Maglia, G. Translocation of linearized full-length proteins through an engineered nanopore under opposing electrophoretic force. *Nat Biotechnol* (2023) doi:10.1038/s41587-023-01954-x.

5. Maglia, G., Heron, A. J., Stoddart, D., Japrung, D. & Bayley, H. Analysis of Single Nucleic Acid Molecules with Protein Nanopores. in 591–623 (2010). doi:10.1016/S0076-6879(10)75022-9.
